# Predictions of Anthropogenic Background PFAS Concentrations in Soil and Relation to Bedrock Lithology and Groundwater Quality

**DOI:** 10.1021/acs.est.5c16810

**Published:** 2026-06-01

**Authors:** Andrea K. Tokranov, Leah M. Santangelo, Joseph D. Ayotte, Sydney M. Welch, Kate Emma A. Schlosser, Jeffrey M. Marts, Anthony F. Drouin, Harrison Roakes

**Affiliations:** † 2928 U.S. Geological Survey, Pembroke, New Hampshire 03275, United States; ‡ 190508 New Hampshire Department of Environmental Services, Concord, New Hampshire 03302, United States; § Sanborn, Head & Associates, Inc., Bedford, New Hampshire 03110, United States

**Keywords:** PFAS, soils, boosted regression tree modeling, fate and transport, groundwater, lithology

## Abstract

Detectable concentrations of per- and polyfluoroalkyl substances (PFAS) have been observed in soils in remote areas and presumably originate from atmospheric deposition. These anthropogenic background concentrations may enable some PFAS to leach to groundwater at levels that exceed regulatory criteria for drinking water. However, anthropogenic background soil concentrations and their connection to groundwater are not well characterized. We developed a boosted regression tree model to predict perfluorooctanesulfonic acid (PFOS) and perfluorooctanoic acid (PFOA) concentrations in shallow soils across northern New England. Low soil pH was the most important predictor of elevated anthropogenic PFOS and PFOA concentrations in background soils, rather than potential PFAS sources, land use, or population density. Total organic carbon (TOC) was also an important predictor for PFOS soil concentrations. Model predictions indicate that 73% of the shallow soils within Maine, Vermont, and New Hampshire exceed New Hampshire’s Soil Remediation Standard for PFOS (0.5 ng/g) and 41% exceed the PFOA standard (0.4 ng/g). Analysis of soil model results and groundwater data suggests that areas with high soil pH are associated with higher groundwater detection frequencies, illustrating how areas with less retention in soil are, conversely, also areas with potentially greater groundwater vulnerability. Further analysis indicates that groundwater may be more vulnerable in calcareous lithologies.

## Graphical abstract

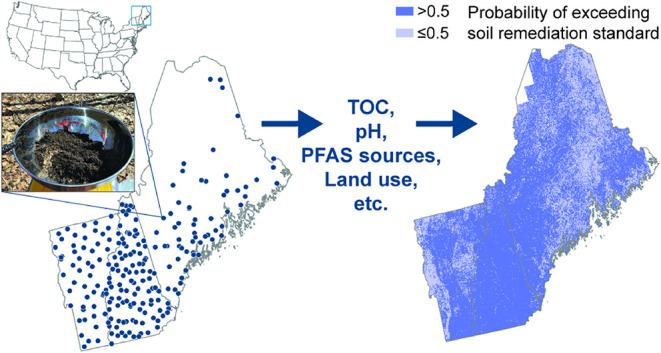

## Introduction

Over the past decade, regulations to protect the public from exposure to per- and polyfluoroalkyl substances (PFAS) have become more numerous as adverse health outcomes have become clearer.^1^ In particular, drinking water has been a primary focus of regulation, with concentration limits protective of public health decreasing substantially over time.^2^ In 2024, the U.S. Environmental Protection Agency (EPA) set the maximum contaminant levels (MCLs) for perfluorooctanoic acid (PFOA) and perfluorooctanesulfonic acid (PFOS) to 4 ng/L in drinking water, but also set the maximum contaminant level goal (MCLG) to zero for both compounds.^3^ These low MCLs have driven questions about how PFAS are introduced to groundwater used as drinking water supplies and whether the MCL can be exceeded even in areas with no identifiable point source of contamination nearby. For example, concentrations of some PFAS in precipitation can exceed the MCLs.^4,5^ In one study, mean PFOS concentrations in precipitation were 20 ng/L at a site in Jackson Hole, Wyoming, that was chosen for its location in undeveloped land and low surrounding population density.^6^ Atmospheric deposition to soils coupled with leaching to groundwater may also theoretically be an important mechanism causing low-level PFAS detections in groundwater. Moghadasi et al.^7^ calculated that soil PFAS concentrations of 5 ng/g could result in concentrations ranging from 2 to 5 ng/L in soil seepage water using the intermediate range of soil-water distribution coefficients for PFOS and PFOA. Recent research by the U.S. Geological Survey (USGS) provides field-based evidence that supports this concept, demonstrating that PFOA in soils with no known point source can seep into shallow underlying groundwater (groundwater near the land surface and near the water table) at concentrations exceeding the PFOA MCL,^8^ although dilution at greater groundwater depths may attenuate the concentrations.

Given that groundwater is an important source of drinking water supply globally and that PFAS in soil may seep to underlying groundwater, understanding the concentration and spatial distribution of PFAS in soils and the ability of soils to retain PFAS is crucial to protect human health. Soil PFAS concentrations at point sources have been intensively investigated in recent years.^9–11^ However, anthropogenic background PFAS concentrations have received less attention until recently, despite the fact that PFAS have been widely found in soils in areas with no known point source.^11,12^ We use the term “anthropogenic background” because the natural background for PFAS considered in this study should be zero, given that these compounds do not occur naturally. We define PFAS anthropogenic background in soil as the low-level concentrations that arise from diffuse sources. More recent work has begun to address the data gap on anthropogenic background in soils. Brusseau et al.^11^ compiled data from over 2500 sites worldwide and found widespread presence of PFAS even at sites expected to represent anthropogenic background (no known point source). Recent regional studies investigating anthropogenic background in soil have found widespread detections in Sweden,^13^ Vermont,^14^ Massachusetts,^15^ Maine,^16^ Michigan,^17^ and Germany.^18^ While data on anthropogenic background concentrations in soil are increasingly becoming available, the factors controlling these concentrations are not well understood. Anthropogenic background soil concentrations are a function of (1) current and historical inputs from wet and dry atmospheric deposition, and (2) retention in the soil depending on a variety of factors. These factors include permeability and drainage characteristics (precipitation rates, hydraulic conductivity, etc.), as well as soil properties that influence PFAS sorption, such as total organic carbon (TOC), protein content, pH, and anion exchange capacity, among others.^19,20^

The objectives of this work were to (1) evaluate the occurrence of PFAS in shallow soils (0–6 in. in depth) that are likely to represent anthropogenic background concentrations in New Hampshire in relation to soil properties and other variables that may affect concentrations, such as nearby potential sources of PFAS, land use, and population density; (2) build a model to predict where elevated concentrations of PFOS and PFOA may be present in shallow soils across northern New England, drawing from additional data available in Maine and Vermont; and (3) analyze model results in relation to lithology and available PFAS data for underlying groundwater. Objective 1 was focused only on New Hampshire soils because the data set is extensive and includes protein and additional sampling depths, among other aspects not included in the Vermont and Maine studies. We hypothesized that both nearby PFAS sources and soil chemistry would affect the PFAS concentrations in soils. A boosted regression tree (BRT) model framework was chosen, given its ability to handle nonlinear trends and correlations between variables.^21^ Predictions of soil concentrations are presented for the three states in northern New England, and preliminary estimates of the mass of PFOA and PFOS in shallow soils are calculated. To our knowledge, this is the first study to predict the anthropogenic background concentrations of PFAS in soil. The results may help prioritize future sampling to evaluate regional groundwater vulnerability and ecosystem toxicity risks and provide actionable information to the public.

## Materials and Methods

### New Hampshire Soils: Study Design, Sampling, and Analysis

Full details on the study design, sampling protocols, analysis, and quality control for the New Hampshire soil samples are available in a publicly available data release^12^ and are described in the Supporting Information (Figure S1). Briefly, the state of New Hampshire was gridded into 100 equal-area grid cells, and a random location was identified and sampled within each cell.^22^ Although the study design was random, access restrictions required modifications for some sampling locations.^12^ Only land uses characterized by the 2016 National Land Cover Database^23^ as forest, shrubland, scrubland, grassland, herbaceous, wetlands, or barren land were sampled to avoid characterization of soil on agricultural and developed lands. Additionally, a 500 m buffer was placed around each known or suspected PFAS source, such as airports, wastewater treatment plants, and fire training areas, to help ensure the locations were not directly impacted by PFAS point sources.^12^ Although there are known widespread atmospheric emission sources of PFOA in the southern part of New Hampshire,^24^ we collected samples in southern New Hampshire because the study objective was to evaluate statewide occurrence and because such large sources may increase the anthropogenic background in the area. Samples were collected from 0 to 6 in. in depth at all 100 locations (Figure 1) and from 6 to 12 in. in depth at 50 locations (refer to the Supporting Information for details). Six sites were additionally sampled down to a maximum of 36 in. in 6 in. increments. The 0 to 6 in. sampling interval was chosen to be consistent with studies in Maine^16^ and Vermont,^14,25^ and because PFAS are known to be retained in organic-rich shallow soils.^19,20^ Samples were analyzed for PFAS (36 compounds; measured as wet weight and reported as dry weight), TOC, pH, moisture content, protein (in the 0–6 in. interval at 91% of the sites), and PFAS after the Total Oxidizable Precursor Assay (in the 0–6 in. interval at 50% of the sites), and soils were visually characterized at each location.

**Figure 1:**
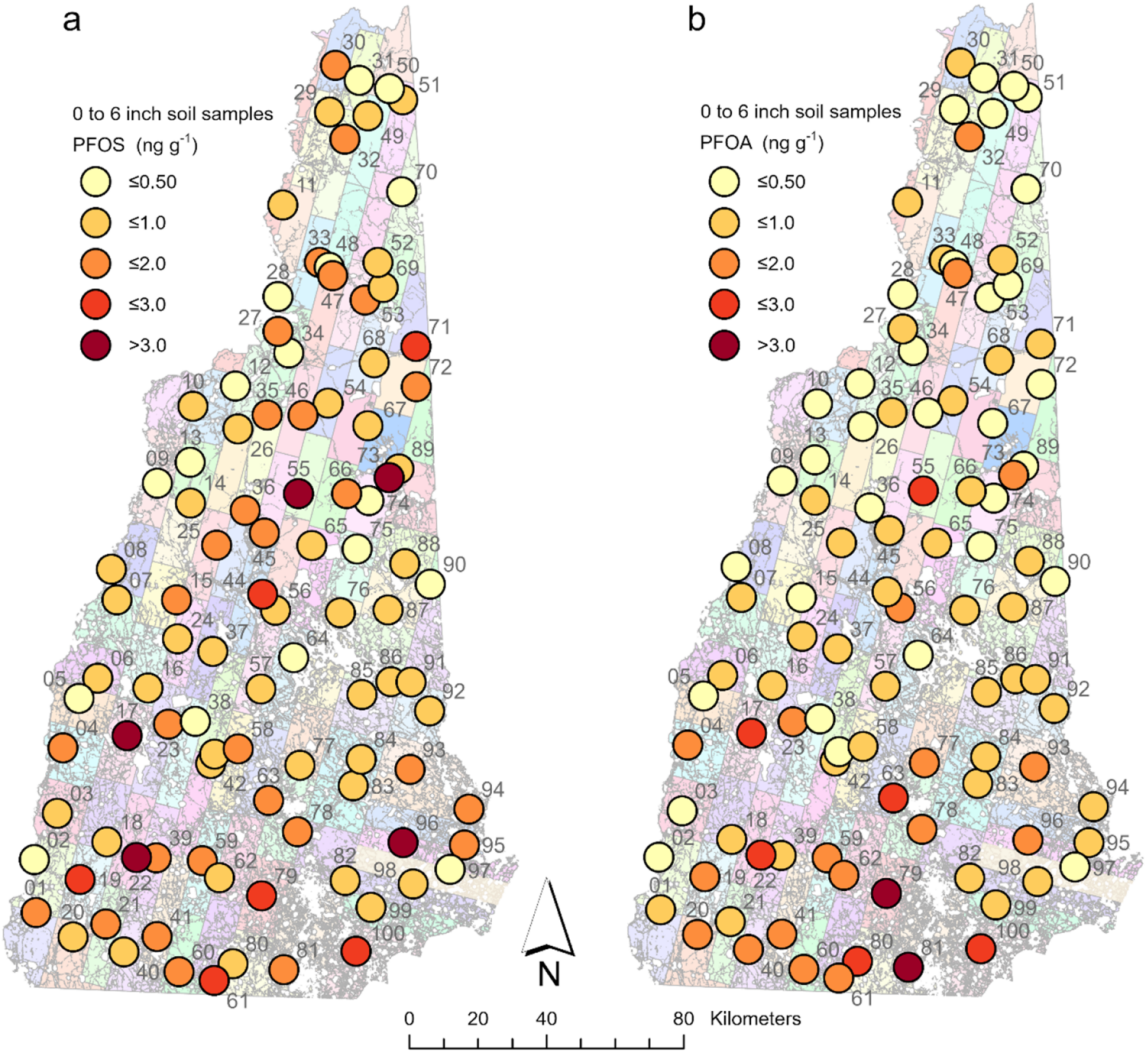
Soil sampling locations (*n* = 100) across the state of New Hampshire, color-coded according to perfluorooctanesulfonic acid (PFOS) (a) or perfluorooctanoic acid (PFOA) (b) concentrations in the 0 to 6 in. sampling interval. Site numbers are displayed next to each sample. Grid cells (*n*=100) are shown in the background (multicolored boxes with gray outlines).

### Data Sets and Variables for Boosted Regression Tree Modeling

PFAS, TOC, and pH data from studies conducted in Maine^16^ and Vermont^14,25^ were compiled along with the data from the 0–6 in. primary samples from New Hampshire^12^ to provide a robust data set of 229 sample points across the northern New England states.^26^ All samples were from the 0–6 in. sampling interval and targeted anthropogenic background concentrations in soil. The methods for location selection in the Maine and Vermont studies resulted in locations that were distributed broadly across those states and included samples from lightly developed properties, such as parks and public greens. Sampling methods for all three states were similar.^12,14,16,25^ Maximum method detection limits for all data were lower than the chosen modeled thresholds for PFOS (0.5 ng/g) and PFOA (0.4 ng/g), which are described below (Table S1).

A total of 36 potential predictor variables were assessed and are given in Table S2. Variables included distance to the nearest potential PFAS source (*n* = 15; e.g., airports, metal coating facilities, fire training areas), soil properties (pH, TOC, fraction of sand, silt, and clay, and hydraulic conductivity), population density, underlying lithology, land cover type, and precipitation, among others. Potential PFAS sources were gathered from the EPA PFAS Analytic Tools.^27^ Note that although this model is for the anthropogenic background in soil and does not predict concentrations at point sources, PFAS sources could contribute to the anthropogenic background in soil through diffuse atmospheric deposition. Measured pH and TOC were used for model training, where available (pH was not available in VT). Predictions could then be made using the pH and organic matter raster data from the Probabilistic Remapping of SSURGO (POLARIS; SSURGO = Soil Survey Geographic Database) data set, which provides TOC and pH values across the study domain.^28^ The organic matter data from POLARIS were converted to TOC using a conversion factor of 1.9.^29^

### Thresholds for Boosted Regression Tree Modeling

The recently published New Hampshire soil remediation standards (SRSs) for PFOS (0.5 ng/g) and PFOA (0.4 ng/g)^30^ were implemented as a threshold to determine where there may be exceedances of the SRS for PFOS and PFOA in shallow soil across northern New England. Although there are SRS values for PFHxS (0.4 ng/g) and PFNA (1.3 ng/g), it was not possible to model these compounds at these thresholds, because almost all data were below the threshold. In New Hampshire, it is anticipated that exceedances of SRS thresholds may require investigation and remediation; however, if the contamination is due to an anthropogenic ambient background condition, then remediation is not required. Therefore, these threshold values were chosen for modeling to help provide actionable information to the New Hampshire Department of Environmental Services and to the public. PFOS and PFOA concentrations from the data sets were set to a binary value of 0 for values less than or equal to the New Hampshire SRS threshold or 1 for values above the standard.

### Boosted Regression Tree Modeling

BRT modeling was done in the R computing environment^31^ using the caret^32^ and gbm^33^ packages with a Bernoulli distribution. All data were used for training the model, given the somewhat limited sample size, and a 10-fold cross-validation was conducted during model tuning to ensure accuracy. To reduce overfitting and improve the generalizability of the models, a simple model within one standard error of the highest accuracy model was chosen. Variables with little model influence (those with a relative influence of <1) were removed from the model so that the final models included only variables important for prediction. A standard threshold of 0.5 was used to evaluate model performance: predicted probabilities less than 0.5 were classified as no exceedance of the New Hampshire SRS. Model performance was evaluated using accuracy, sensitivity (true positive rate), specificity (true negative rate), and the area under the receiver operator characteristic (ROC) curve. Model uncertainty was characterized by bootstrapping the training data and estimating 95% confidence intervals for predicted probabilities based on variability across 100 bootstrap iterations. Microsoft Copilot was selectively employed to debug code for figures and to generate code for the calculation of confidence intervals and SHapley Additive exPlanation (SHAP) values. All of the code was reviewed, tested, and validated by the authors to ensure correctness and reproducibility. The model code, training data, and prediction output can be found in the associated data release.^26^

## Results and Discussion

### New Hampshire Data

At least one of the 36 PFAS compounds analyzed was detected in every sample taken from 0 to 6 in. and 6 to 12 in. in depth below the land surface, indicating the pervasive occurrence of PFAS in areas not known to have impacts.^12^ Of the 16 samples taken at depths deeper than 12 in., 14 had at least one compound concentration above the method detection limit.^12^ For the 0 to 6 in. samples, a maximum of 21 PFAS were detected within a single sample, and detection frequencies (Figure S2) were highest for PFOS and PFHpA (100%), PFNA (99%), PFOA (96%), and PFPeA (93%). Detection frequencies were generally low for compounds other than perfluoroalkyl acids, such as 6:2 fluorotelomer sulfonate (2.0%) and *N*-ethyl perfluorooctanesulfonamidoacetic acid (16%). Implementation of Regression on Order Statistics using the NADA program^34^ for 0 to 6 in. samples indicates that the highest median PFAS concentration was 0.96 ng g^–1^ (mean = 1.2; Standard Deviation [SD] = 0.91) for PFOS, followed by 0.76 ng g^–1^ (mean = 0.90; SD = 0.72) for PFOA. For the 6 to 12 in. interval, the highest median PFAS concentration was 0.59 ng g^–1^ (mean = 0.82; SD = 0.76) for PFOA, followed by 0.45 ng g^–1^ (mean = 0.64; SD = 0.66) for PFOS. These concentrations are higher than what has been observed in Vermont and Maine, as has been documented by McIntosh et al., 2025.^15^ It has been suggested that these differences may be driven by population and land use,^15^ although we find only weak correlations with these parameters as discussed below.

PFAS concentrations typically decreased with an increase in soil depth in New Hampshire. The 0 to 6 in. depth interval had significantly higher concentrations than the 6 to 12 in. depth interval (*p* < 0.05, one-sided Wilcoxon signed rank test, Table S3) for every perfluoroalkyl acid with detection frequencies of >20% in both depth intervals. This aligns with previous work that has also reported concentration reductions with depth, although these studies were at sites with known PFAS releases^11,35–39^ and not locations representative of anthropogenic background. Depth profiles varied by the compound. Depth ratios were calculated as the concentration in the 0 to 6 in. interval divided by the concentration in the 6 to 12 in. interval to evaluate trends. The depth ratios were generally highest for compounds with longer chain lengths compared to compounds with shorter chain lengths (Figures S3 and S4), indicating that retention in the shallow zone is driven by greater sorption for compounds with longer chain lengths. Because TOC is well-known to enhance sorption to soil,^20^ PFAS residuals (difference between observed and predicted values) were calculated after regressing each PFAS compound on TOC. This allows for an evaluation of whether there are other features besides TOC that are important to PFAS retention in the soil. A one-sided Wilcoxon signed rank test indicated that the 0 to 6 in. depth interval had significantly higher concentrations than the 6 to 12 in. depth interval (*p* < 0.05, Table S3) for PFBA and long-chain-length compounds PFNA, PFDA, PFUnA, PFDoA, PFTrDA, and PFOS based on this residual analysis. This indicates that other factors besides TOC affect PFAS sorption in shallow soil, particularly for long-chain-length compounds.

One or more PFAS compounds exhibited significant Spearman correlation coefficients (*p* < 0.05) with protein concentration and population density (positive coefficients), and agricultural land use (within a 1 km buffer around the sites) and pH (negative coefficients) (Figure S5). Population density was not well-correlated with PFAS (except for PFOA), but several compounds exhibited weak correlations with population density after the implementation of TOC residuals analysis. These various confounding factors indicated a need for additional analysis to better understand the drivers behind the spatial distribution of PFAS. Therefore, BRT modeling was undertaken to (1) determine which variables are most important in predicting PFOA and PFOS concentrations and (2) make predictions of elevated PFOA and PFOS concentrations in shallow soil across northern New England.

### Boosted Regression Tree Model

Accuracies derived from 10-fold cross-validation using out-of-fold predictions were 0.78 (95% Confidence Interval [CI]: 0.72–0.83) for PFOS and 0.78 for PFOA (95% CI: 0.71–0.84) and the area under the ROC curve (AUC) was 0.85 (95% CI: 0.79–0.91) for PFOA and 0.83 (95% CI: 0.77–0.90) for PFOS. Using the full data set, the final PFOS and PFOA models had overall accuracies of 0.80 for PFOS and 0.85 for PFOA and AUC values of 0.91 for PFOA and 0.89 for PFOS, indicating good model performance (Table 1, and Figure 2). By state, overall model accuracies for the full training data were similar, although slightly lower in Vermont for PFOS. In general, sensitivity was higher, and specificity was lower for New Hampshire and Vermont, while the opposite was true for Maine (Figure 2, and Table 1), likely because most of the data were below SRS thresholds in Maine for both PFOS and PFOA. The observed PFOS class imbalance in New Hampshire, and to a lesser extent Vermont, may contribute to the high model sensitivities and low specificities (Table 1), which indicates a tendency to overpredict PFOS threshold exceedances in those states. In contrast, the PFOA model had more balanced sensitivities and specificities across states. Similarly, overall specificity and sensitivity values for the whole data set were also reasonable. All model iterations, as well as the final selected models, indicated that soil pH was the strongest predictor of whether PFOS or PFOA concentrations would exceed the New Hampshire SRS thresholds. TOC was the second most important predictor for PFOS. Despite class imbalance, SHAP values showed that soil pH remained the strongest predictor, whether using the full study area background or state-specific background, for both PFOS and PFOA (Figure S6).

**Figure 2:**
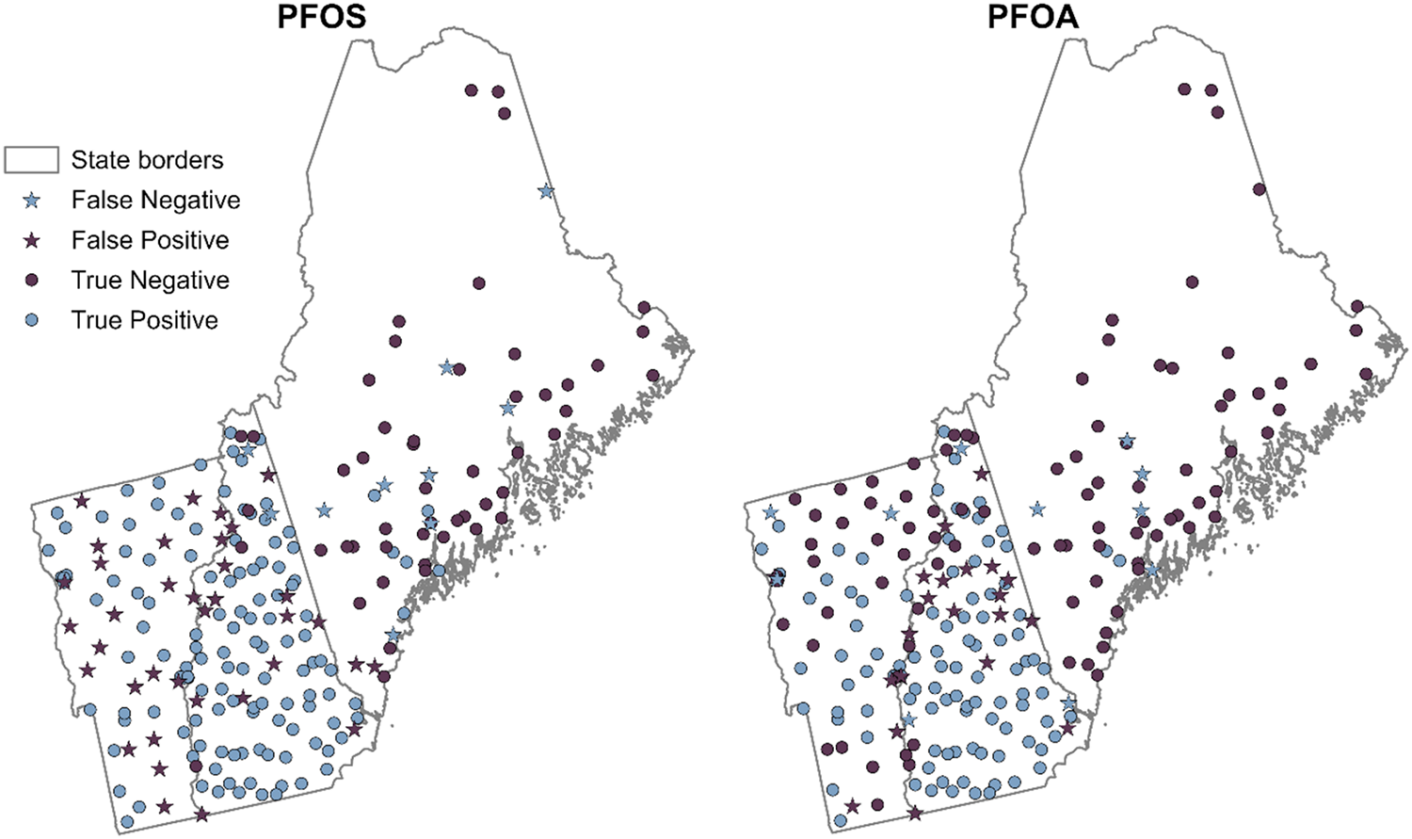
Training data model results for perfluorooctanesulfonic acid (PFOS) and perfluorooctanoic acid (PFOA).

**Table 1:**
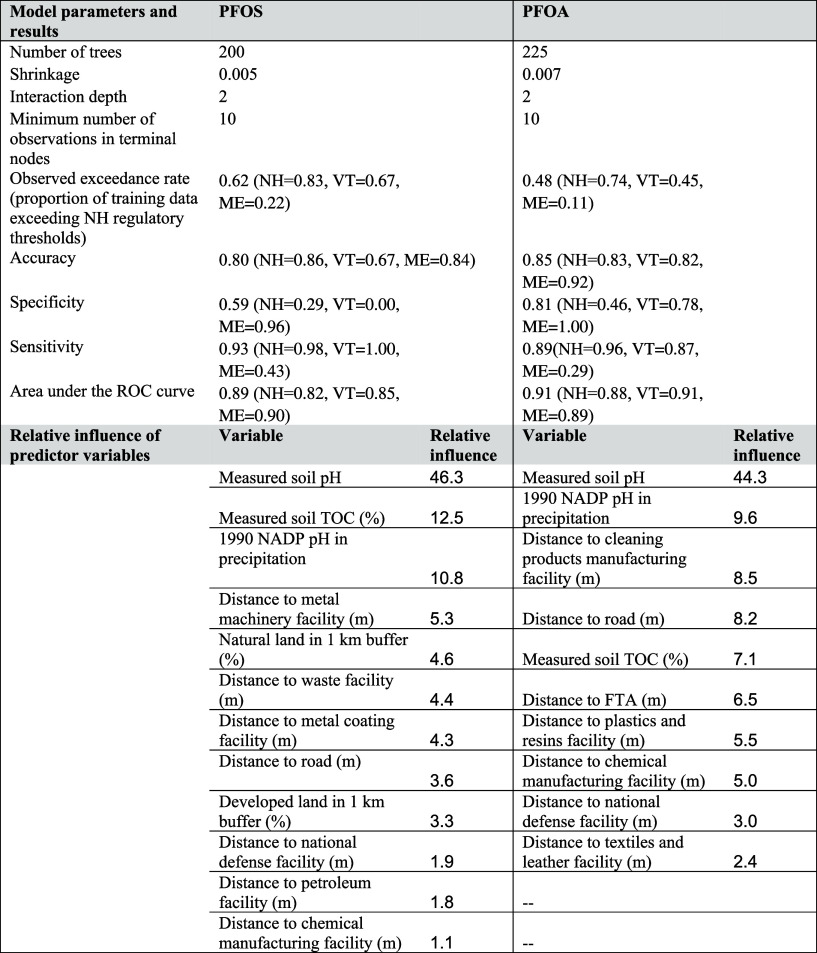
Model Results for Perfluorooctanesulfonic Acid (PFOS) and Perfluorooctanoic Acid (PFOA)^a^

a Thresholds were 0.5 ng/g (PFOS) and 0.4 ng/g (PFOA), respectively. Metrics reported for the full set of training data. FTA = Fire training area; NADP = National Atmospheric Deposition Program; ROC = Receiver Operator Characteristic; TOC = Total organic carbon; ME = Maine; NH = New Hampshire; and VT = Vermont.

Soil pH had a relative influence far beyond soil TOC in both models (Table 1), indicating that pH contributes more to the model variance reduction than TOC. Lower pH and higher TOC were linked to higher probabilities of SRS exceedances (Figures S7 and S8). It is well-established that pH and TOC influence PFAS sorption, with PFAS sorption often increasing with increasing TOC and decreasing pH.^19,20^ Decreasing pH results in greater protonation of the soil surfaces, which can enhance electrostatic interaction and also modify the charge of organic matter functional groups and metal oxides.^40,41^ Organic matter has long been shown to affect PFAS transport through hydrophobic interactions, although it has been repeatedly shown that it cannot be used to predict sorption on its own.^19,40^ The prominence of pH for both models and TOC for the PFOS model aligns with a critical analysis by Li et al., 2018,^42^ where it was shown that multiple regression with pH and organic carbon was generally able to explain *K*_d_ value variation for PFOS and PFOA.^42^

Interestingly, the map of precipitation-weighted mean pH in atmospheric deposition from 1990 ^43^ had predictive power in both models. In 1990, the Clean Air Act was amended to address sulfur dioxide and nitrogen oxide emissions to curtail the observed pH drop in precipitation.^44^ The importance of this variable in both models suggests that it could act as a proxy for regional wind and general atmospheric depositional patterns. For example, we hypothesize that low-pH atmospheric deposition could correlate with historical deposition of PFAS if historical PFAS emissions and fossil fuel plant emissions were sourced from similar locations and were affected by similar atmospheric transport and depositional mechanisms.

Distances to various potential sources of PFAS had some model influence and were retained in the final models, but were, overall, not as important as soil pH. All partial dependence plots showed decreasing PFOS/PFOA probability with increasing distance away from each potential source (Figures S7 and S8). The potential PFAS sources identified as important predictors differ between the PFOS and the PFOA models.

For PFOS, the distance to metal coating, machine metal, waste, national defense, petroleum, and chemical manufacturing facilities was important. PFOS has historically been used as a wetting agent for metal plating, particularly chrome plating, and as a mist suppressant for metal electrowinning and in the manufacture of metals,^45^ likely explaining the associations in the model. Similarly, PFAS are used in chemical manufacturing as a processing aid, as inert media for reactions, and as solvents, among other uses.^45^ National defense sites include the Pease Air National Guard Base, Loring Air Force Base, and several defense training sites and Army and Air National Guard locations. Besides extensively documented use in aqueous film-forming foam (AFFF),^46–48^ PFAS may also be found in fluorinated oxidizers for pyrotechnics or in rocket propellants and jet fuel, among other uses relevant to defense sites.^49^ Petroleum facilities may use PFAS for refining products or may have AFFF onsite for petroleum fire emergencies.^45^ Waste facilities include wastewater treatment plants, solid waste landfills, hazardous waste treatment and disposal facilities (excluding remediation sites), and solid waste incinerators. Wastewater treatment plants are known to emit PFAS to the atmosphere,^50^ and in the air surrounding a wastewater treatment plant in Ontario, Canada, PFOS has been shown to be higher in concentration than PFOA.^50^ Atmospheric emissions from landfills are typically dominated by fluorotelomer alcohols (FTOHs), which can degrade to perfluoroalkyl carboxylates like PFOA over time.^51,52^ In contrast, PFOS is typically not observed at high concentrations in landfill emissions because of its low vapor pressure.^51^ Notably, the distance to waste facilities was an important predictor for PFOS but not for PFOA, suggesting air emissions from wastewater treatment plants and not from other waste facilities may be of more significance for PFOS. This is supported by the greater number of wastewater treatment facilities identified by NAICS code 221320 (*n* = 199) compared with the number of landfill facilities (NAICS code 562212, *n* = 44) within the study area.

For the PFOA model, the distance to chemical manufacturing and national defense sites was important, similar to the PFOS model. In addition, the PFOA model found distance to cleaning products manufacturing facilities, textiles and leather facilities, plastics and resins facilities, and fire training area facilities to be important predictors. PFAS are often added to cleaning products to lower surface tension and impart stain/water-resistant properties.^49^ PFAS have been frequently used as coatings on textiles and leather to impart stain- and water-repellent properties,^49^ and PFOA-related chemicals have been used to prevent foaming during sulfur-based dyeing processes.^45^ The importance of the plastics and resins facilities and textiles facilities for the PFOA model was unsurprising, given the well-known PFOA-dominated emissions from a manufacturing facility producing polytetrafluoroethylene-coated fabrics in New Hampshire and its use during emulsion polymerization for fluoropolymer production.^24^ Finally, distance to fire training areas was important to the PFOA model and may be driven by PFAS-containing AFFF usage.

The percentage of natural land within a 1 km radius around the sampling location was generally inversely related to the probability of PFOS threshold exceedance (Figure S8), except for the highest natural land use percentages, which had an increased probability, suggesting that there may be factors unaccounted for by the model. The percentage of developed land within a 1 km radius around the sampling location had a positive relationship with PFOS probabilities, as expected (Figure S8). This aligns with previous work in groundwater and tapwater, which also found greater probabilities of PFAS detections in urban areas.^53–55^ These trends indicate that other sources of PFAS besides those captured by the documented potential sources included in this modeling may be important. Interestingly, we did not find population density (a parameter correlated with developed land; Figure S5) to be an important model variable, in contrast to other soil studies.^13,14,56^ However, these studies noted correlations with population density but did not attempt to differentiate the effects of population from sources, land use, or other variables, as was done in this modeling approach. The lack of importance for population density in the model indicates that soil properties, potential PFAS sources on the landscape, and land use, among other parameters, may be more important in explaining PFAS threshold exceedances.

The distance to the nearest road was included in the model as a potential predictor variable because non-native material may be introduced during road construction, the nearby soil structure may be disturbed,^57^ and trash litter may introduce PFAS material to the surrounding landscape. In both PFOS and PFOA models, a greater distance from the road was surprisingly associated with higher probabilities of threshold exceedances. This may suggest that the soil structure is disturbed closer to roads. Roads are typically designed to be well-drained, and the adjacent soil may be non-native,^57^ which we hypothesize increases hydraulic conductivity and therefore promotes increased flushing of PFAS out of the soils. Additionally, there may be increased water volumes from road runoff due to impervious cover,^57^ which may also increase soil flushing.

Overall, the model was heavily influenced by the soil pH, and TOC to a more limited degree for PFOS, with only relatively small contributions from other variables, including distances to potential PFAS sources. McIntosh et al., 2025,^15^ suggested that the PFAS profiles in northern New England are reflective of atmospheric deposition, an interpretation that this model supports. If local sources were the primary controlling factor for PFOS and PFOA threshold exceedances, then the models should theoretically identify PFAS sources as more important model predictors. One possible interpretation is that local, regional, and more distant air sources of PFAS blend and transport across the region, resulting in a blanketing effect on the deposition.

The potential implication and irony of these modeling outcomes are that areas prone to high soil PFAS levels (with low pH and high TOC) may be protective of groundwater because of soil retention, whereas areas prone to low soil PFAS levels (with high pH and low TOC) may indicate areas where PFAS are more readily transported downward to the water table. This testable hypothesis is discussed in more detail below.

## Predictions of Anthropogenic PFOS and PFOA Background in Shallow Soil Across Northern New England

Predictions of PFOS and PFOA SRS threshold exceedances in soil indicate that most of the area in northern New England will exceed SRS thresholds for PFOS (Figures 3, and S9). Specifically, 73% (95% confidence interval [CI]: 53–96%) of the study area is predicted to exceed the SRS threshold for PFOS, while 41% (95% CI: 19–73%) of the area is predicted to exceed the SRS threshold for PFOA. Note that the model predicts areas of elevated anthropogenic background concentrations in soil and is therefore not representative of concentrations in locations with known PFAS release (e.g., a field with applied biosolids, or a site with AFFF release). For Vermont, Maine, and New Hampshire, PFOS soil thresholds were predicted to be exceeded in 72% (95% CI: 43–98%), 66% (95% CI: 46–94%), and 97% (95% CI: 86–100%) and PFOA soil thresholds were predicted to be exceeded in 46% (95% CI: 32–62%), 25% (95% CI: 6–70%), and 89% (95% CI: 53–97%) of each state, respectively. New Hampshire had the highest rate of exceedance for both PFOS and PFOA, in line with the high observed detection frequency from the New Hampshire soil occurrence study, which was 83% for PFOS and 74% for PFOA.

**Figure 3:**
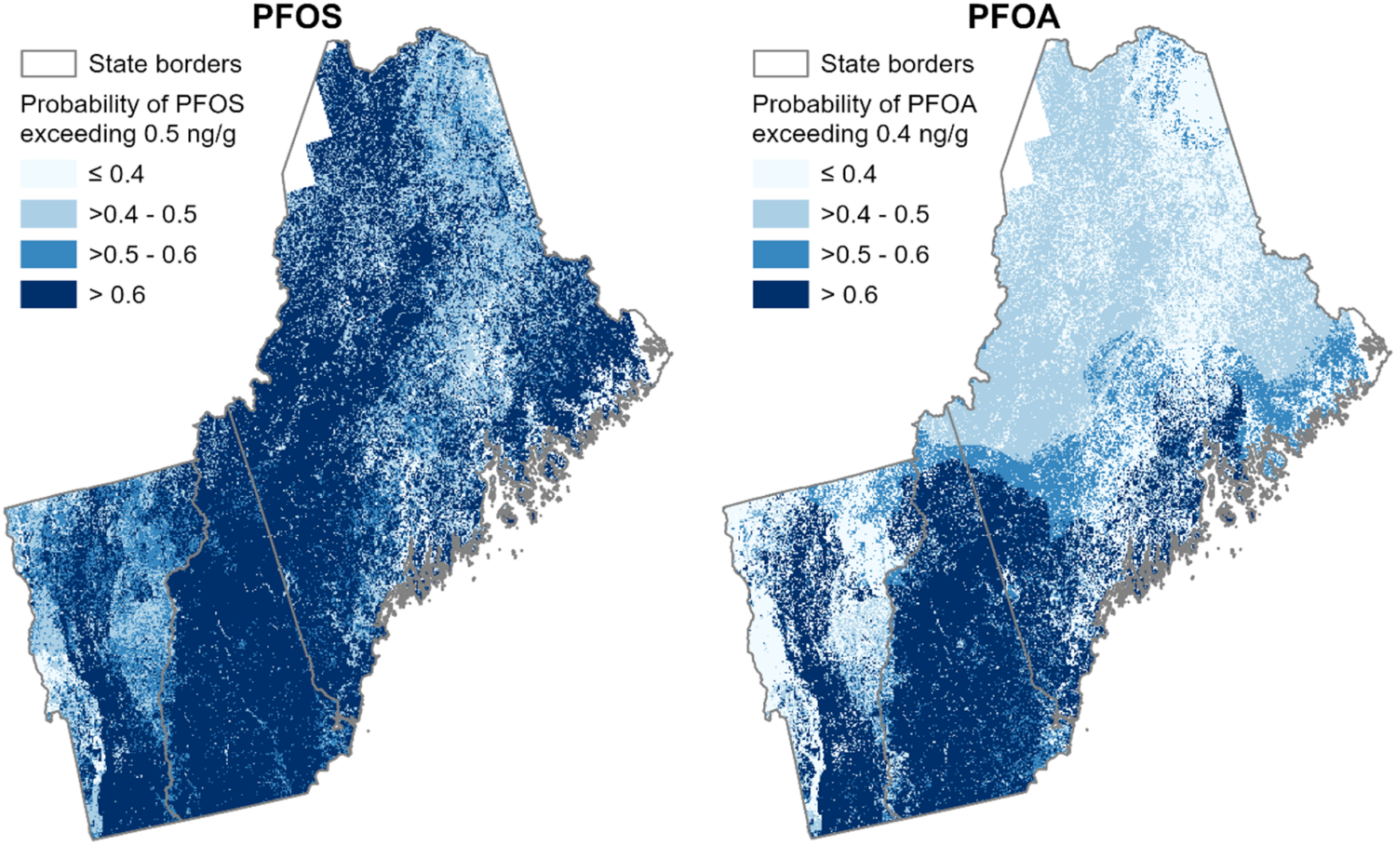
Model predictions of perfluorooctanesulfonic acid (PFOS) and perfluorooctanoic acid (PFOA) concentrations exceeding the New Hampshire Soil Remediation Standards (SRS) of 0.5 ng/g and 0.4 ng/g in soil, respectively. Each grid cell has a predicted probability: predicted probabilities that exceed 0.5 are classified as exceeding the SRS limit and correspond to the darkest two blue colors.

The mass of PFAS predicted to be in the top 0 to 6 in. layer of soil can be conservatively estimated by assuming the areas predicted to be over the threshold have soil concentrations of 0.5 ng/g for PFOS and 0.4 ng/g for PFOA, and that areas beneath the threshold have concentrations of 0 ng/g. These represent the lower bounds of the prediction categories (exceeded SRS concentration or below SRS concentration) and therefore result in conservative and preliminary estimates. Using depth-weighted values for bulk density from 0 to 15 cm depth in each grid cell from the POLARIS data set^28^ to convert from ng/g to ng/cm^3^, the PFOA mass residing in shallow soil in each state absent local sources is estimated to be 780 kg (95% CI: 540–1100 kg) in Vermont, 1400 kg (95% CI: 330–3,800 kg) in Maine, and 1400 kg (95% CI: 870–1600 kg) in New Hampshire (3600 kg across all three states; 95% CI: 1700–6500 kg), and the PFOS mass is predicted to be 1500 kg (95% CI: 910–2,100 kg) in Vermont, 4500 kg (95% CI: 3100–6500 kg) in Maine, and 2000 kg (95% CI: 1700–2000 kg) in New Hampshire (8000 kg across all three states; 95% CI: 5700–11,000 kg). This aligns well with PFOA atmospheric deposition estimates from Thackray et al., 2020, which predicted between 930 and 8500 kg of PFOA deposition in Vermont, New Hampshire, and Maine from 1960 to 2015 from nonlocal sources (detailed output of published material obtained through personal communication).^58^ To contextualize these preliminary estimated masses, they are the hypothetical equivalent mass of 900 trillion liters (PFOA; 900 billion m^3^; 95% CI: 430–1600 trillion liters) or 2000 trillion liters (PFOS; 2000 billion m^3^; 95% CI:1400–2800 trillion liters) of water if the water had a uniform concentration at the EPA MCL of 4 ng/L. If the soil were to leach PFAS with the exact concentration of the EPA MCL of 4 ng/L, and assuming an average annual normalized groundwater recharge rate of 21 in. per year,^59^ these preliminary volumes represent approximately 13 years (PFOA; 95% CI: 6–23 years) and 28 years (PFOS; 95% CI: 20–39 years) of recharge over the combined areas of Vermont, New Hampshire, and Maine. Whether the underlying groundwater will be contaminated by the PFOS and PFOA predicted to be stored in these soils will be dependent on numerous factors, including soil pH, TOC, precipitation rates, soil structure, unsaturated zone depth, hydraulic conductivity, and a variety of other factors. However, these estimates indicate a substantial reservoir of PFAS exists in the shallow soil in northern New England.

The large masses of PFOS and PFOA predicted to exist in the shallow soil in northern New England are of potential concern for future water quality. Although the predictive map (Figure 3) may be useful in identifying areas where exceedances of the SRS might be expected in shallow soils, these exceedances are not necessarily indicative of greater potential for groundwater contamination. Specifically, as suggested in the previous section, areas with greater predicted soil concentrations are often areas of increased PFAS retention associated with low soil pH and high soil TOC values. Conversely, areas with lower predicted soil concentrations are often areas of decreased PFAS retention associated with high soil pH and low soil TOC values and are hypothesized to have had historically greater transport of PFAS from soil to groundwater.

We tested the hypothesis that high soil pH and low soil TOC are associated with greater groundwater vulnerability using groundwater data from New Hampshire’s Drinking Water and Groundwater Trust Fund (DWGTF) study,^60^ which sampled for PFAS in private wells (overburden and bedrock wells) used for drinking water across the state between 2019 and 2020 (*n* = 435). PFOS and PFOA detection frequencies in groundwater were calculated by bins of low and high soil pH and TOC from the POLARIS data set,^28^ using the median value to define the cutoff points (4.46 for pH, 4.76 for TOC). For both PFOS and PFOA, the groundwater detection frequency was highest for the high soil pH and low soil TOC bin (Figure 4a,b), although only PFOS had a significant (<0.05) chi-squared *p* value. TOC and pH were evaluated individually (Figure 4c–f) as well. Notably, while the soil pH bins produced a significant chi-squared *p* value for PFOS (*p* < 0.05) and to a lesser degree for PFOA (*p* < 0.1), TOC did not produce significant chi-squared *p* values for PFOS and only a weak *p* value for PFOA (*p* < 0.1), which supports the hypothesis that high soil pH results in greater groundwater vulnerability and is consistent with the model results that identify pH as the variable with greatest influence. For each groundwater well, the associated model SRS probabilities and soil pH and TOC values were also plotted (Figure 4g–h) and highlight how SRS probabilities are statistically lower in areas of high soil pH and low soil TOC for both PFOS and PFOA (*p* < 0.05 based on Kruskal–Wallis with posthoc Dunn’s test). While the results are not conclusive for PFOA, they are consistent with the hypothesis that areas with high soil pH and potentially low soil TOC may have had greater transport of PFOS from soil to groundwater. Therefore, areas that look “clean” with low concentrations of PFAS in the shallow soil may in fact be areas more prone to groundwater contamination due to enhanced downward transport of some PFAS with percolating soil water.

**Figure 4:**
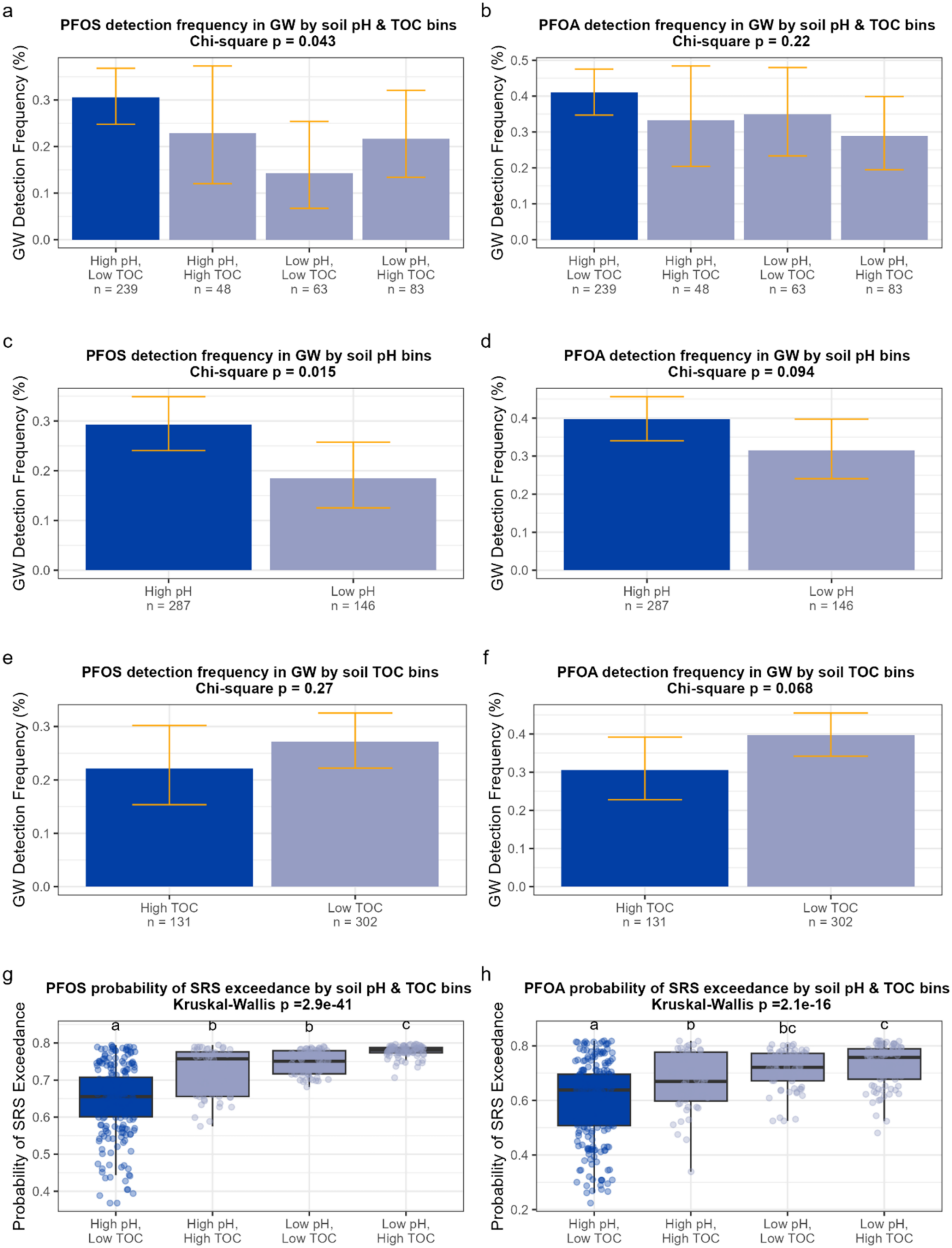
**(a–f)** Groundwater (GW) detection frequency of perfluorooctanoic acid (PFOA) and perfluorooctanesulfonic acid (PFOS) by bins of low (<4.46) and high (≥4.46) soil pH and low (<4.76) and high (≥4.76) soil total organic carbon (TOC). The number of samples in each category is reported as n values on the *x*-axis. Chi-squared p values are reported for each plot. Confidence intervals are shown in orange. **(g–h)** Boxplots of probability of exceeding the New Hampshire Soil Remediation Standards (SRS) by bins of low and high soil pH and soil TOC using the same classification as panels a b. SRS values, as well as soil pH and soil TOC values, are from the grid cell associated with each groundwater well for direct comparison with panels a–f. Kruskal–Wallis *p* values are reported in the plot title, and posthoc Dunn’s test results are reported as letters above the boxplots. Common letters indicate no significant difference at *p* < 0.05. Boxes extend to the first and third quartiles, whiskers extend to 1.5 times the interquartile range. Data are overprinted on the plot.

One potentially complicating factor is the presence of a manufacturing facility with a known history of PFOA emissions south of Manchester, New Hampshire, in the same areas where soil pH is high and TOC is low.^24^ A multipronged approach was taken to investigate this (refer to the Supporting Information for details), and we found that the trends remain comparable to Figure 4 for all three boundary conditions under consideration for both PFOA and PFOS (Figures S10–S12).

## Relationships between Lithogeochemistry and Soil and Groundwater PFAS

We also investigated bedrock lithology as a factor that may influence soil chemistry,^61^ PFAS transport, and ultimately may affect underlying groundwater. The lithogeochemistry of bedrock often influences the overlying soil geochemistry because many soils are developed on sediment derived from underlying bedrock.^62^ Although the lithogeochemical groups were not important to our soil model because of the overwhelming influence of measured soil pH and moderate influence of TOC, they can directly influence soil pH and TOC. Additionally, lithology may also be important for the fate and transport of PFAS from soil into underlying groundwater because hydraulic conductivity can be directly related to lithology (for example, some fractured bedrock aquifers are more transmissive than others).^24,63^ Using modified lithogeochemical groupings from previous work,^61,63^ it is apparent that calcareous rocks in particular had distinct geochemical and contaminant signatures. For example, the median soil pH was high, and the median soil TOC was low (Figure S13) within the calcareous rock units compared to the rest of the study area. The water pH in calcareous rock aquifers is typically elevated due to the presence of calcium carbonate or calcite. The soil prediction rasters for PFOS and PFOA also indicated lower median probabilities for soil SRS exceedances within rock units described as calcareous (Figure S14). Finally, underlying groundwater samples in New Hampshire show the highest detection frequencies for PFOS (49%) and PFOA (66%) in areas containing calcareous rock (Figure 5). Again, this trend did not change when removing wells around a known manufacturing facility, although differences were less statistically significant for the largest boundaries considered (Figures S15–S17). These results suggest that the underlying bedrock lithology can influence PFAS transport from soils into underlying groundwater and that calcareous rocks in this study area may be associated with higher soil pH and higher PFAS detection frequency in groundwater. In New Hampshire, metasedimentary rocks from calcareous marine protoliths are located primarily in the southeastern region of the state and are largely composed of the Berwick, Eliot, and Kittery Formations, and in this analysis are all grouped as “Calcareous Rocks”.^61^ In many cases, these rocks have been tilted on edge so that the original bedding planes (or metamorphic compositional layers) are now vertical and contain fractures that can transmit water vertically downward, possibly resulting in a direct pathway to water supply wells that capture bedrock groundwater. Previous work also found that the Berwick Formation in particular is susceptible to methyl *tert*-butyl ether^64^ (MTBE) and arsenic^65^ contamination. The New Hampshire Department of Environmental Services has also previously suggested that different lithologies within the Berwick exhibit differences in PFAS fate and transport, although a formal analysis was not done.^66^ Our analysis suggests that groundwater may be more vulnerable in calcareous lithologies that are characterized by flow through fractured rock, low soil TOC, and high soil pH. More broadly, we show how bedrock lithology may control soil chemistry and contaminant transport into groundwater, which is an area of study that has received relatively little attention but can be of great importance to how and where PFAS move in soil and groundwater. Although beyond the scope of this study, additional work could delve into the complexities of the intersection of soil properties (pH and TOC) and soil development, which is dependent on multiple factors, including climate, vegetation, biological activity, and topography.

**Figure 5:**
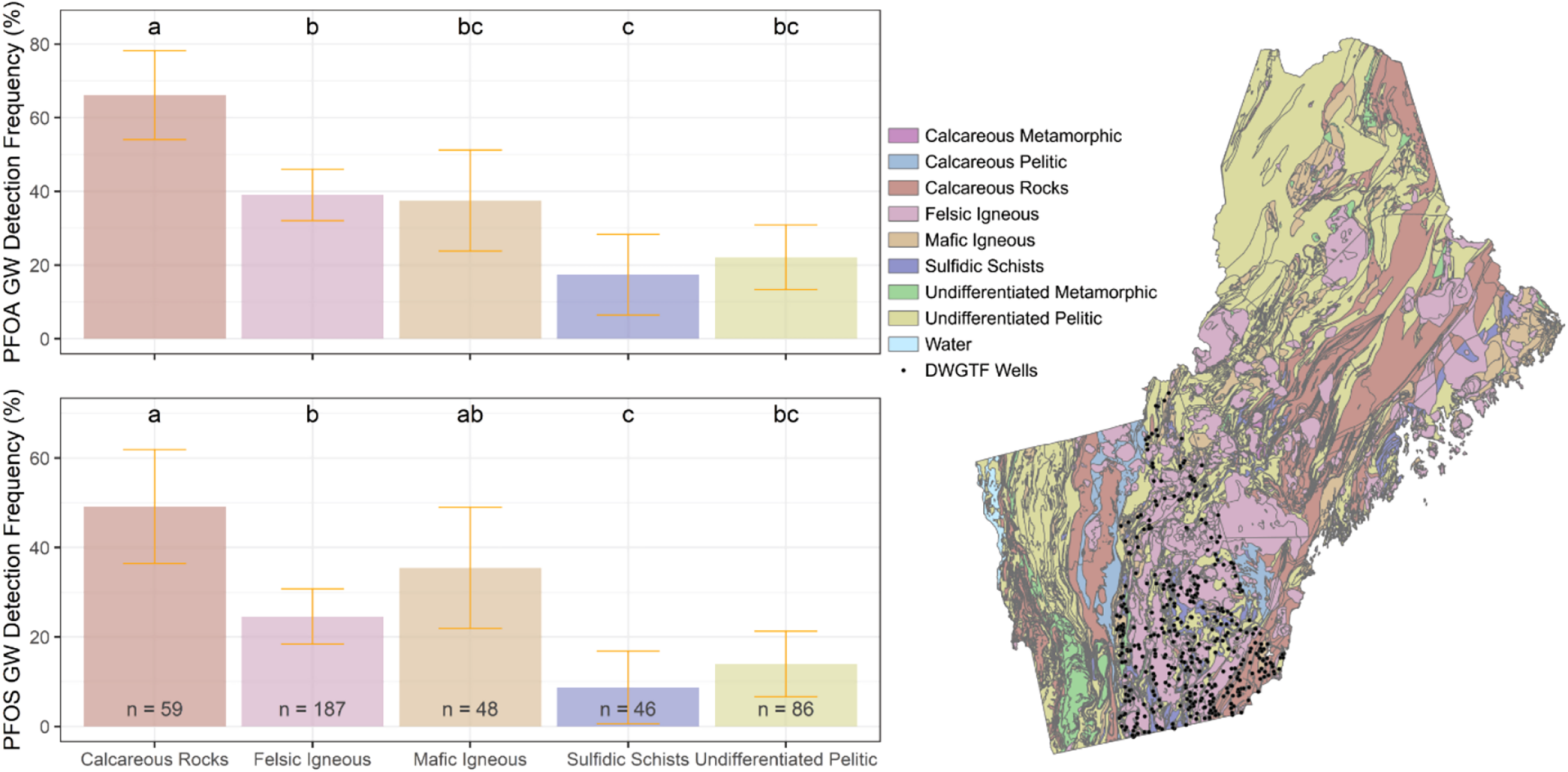
Groundwater (GW) detection frequencies for perfluorooctanesulfonic acid (PFOS) and perfluorooctanoic acid (PFOA) grouped by lithology as shown on the map to the right. Orange bars represent confidence intervals. The results for “undifferentiated metamorphic” were removed because there were only 9 samples within that category. Pairwise proportion tests were used to identify significant differences (*p* < 0.05) and are denoted by different letters at the top of each bar. On the map, wells in black are from New Hampshire’s Drinking Water and Groundwater Trust Fund (DWGTF) study.^60^ Note that there are fewer lithology categories in the bar plot than in the map on the right, because the DWGTF wells do not overlap with all lithologies.

## Limitations and Implications

This study investigates parameters that potentially influence the anthropogenic background concentrations in soil and presents a predictive model of anthropogenic background soil PFAS concentrations in unsampled areas. These predictions do not encompass urban or agricultural land uses or other places known to have PFAS directly released to the soil; therefore, it would be expected that soil PFAS concentrations in those areas would be modified by those land uses. For example, although fire training areas were a weak predictor in the PFOA model, the model estimates their influence only at distant locations, where they contribute to anthropogenic background concentrations. It does not predict concentrations in the immediate vicinity of fire training areas. This is because our input training data is purposefully biased toward locations with no known releases, and so the model predictions are also assuming no known release. Note that one limitation of the model is incomplete information on potential sources available in the EPA PFAS Analytic Tools. For example, the documentation acknowledges that FTA sites may be missing or misidentified.^27^ Improved documentation of potential sources, as well as distinction between known and suspected sources, could enhance future modeling efforts. Further, the currently available training data set is relatively small, and a larger data set would likely improve model accuracy. An independent holdout data set would also enable model validation. Additionally, predictions were made using the POLARIS data set for pH and TOC, which may not be accurate on a local scale. Further information on soil properties, such as anion exchange capacity and mineralogy, may also improve model performance. Finally, although all three studies conducted in Maine, Vermont, and New Hampshire were focused on assessing anthropogenic background in soils, there is always the possibility that some of the soil sampled was locally contaminated.

This modeling suggests that anthropogenic background concentrations in soil are primarily controlled by soil properties and their effect on the retention of PFAS to solid surfaces, rather than proximity to point sources of PFAS that become diffuse at larger scales and are therefore weak predictors of soil PFAS concentration. Our results support the hypothesis that locations with high anthropogenic soil background concentrations often occur where shallow soil retained PFAS (i.e., limited transport to groundwater) and locations with low anthropogenic soil background concentrations may occur where PFAS have percolated through the top layer of soil and into groundwater. In other words, areas with low predicted soil concentrations are potentially areas of greater groundwater vulnerability. Therefore, strategic groundwater monitoring may focus on areas with low soil PFAS concentrations, high soil pH, and low soil TOC, while optimal soil sampling design may focus on the opposite types of locations characterized by low soil pH and high soil TOC. Additional work would be needed to assess the transport of PFAS from background soils, determine to what extent PFAS seepage through the unsaturated zone may contribute to concentrations in groundwater, and establish whether underlying groundwater flow could decouple these soil-to-groundwater associations.

Future studies could help determine whether these findings are more broadly applicable beyond our study area. New England is dominated by (although not limited to) soils derived from crystalline bedrock that are low in pH and TOC, but these findings may transfer to other parts of the United States and worldwide where similar geologic settings exist. This research reveals how underlying bedrock lithology can be interconnected to soil properties and groundwater vulnerability. Additional research on this topic may help facilitate larger-scale modeling if certain types of bedrock have the propensity for PFAS contamination because of their chemistry and hydrologic properties.

## Supplementary Material



## Data Availability

Associated data releases: 10.5066/P9KG38B5, 10.5066/P1K5IUJ6.

## Notes

Additional text on materials, methods, study design, sampling information, analytical information, quality assurance/quality control, data quality evaluation for New Hampshire soil data, sampling design for Maine and Vermont, calculation of PFOS and PFOA mass residing in northern New England states, and analysis of the effect of a manufacturing facility with known PFOA emissions, and lithogeochemical groupings; matrix spike recoveries of New Hampshire soil data (Figure S1); detection frequency in shallow New Hampshire soils (Figure S2); concentration depth ratios for New Hampshire soil data (Figure S3); boxplots of concentration depth ratios for New Hampshire soil data (Figure S4); Spearman correlation plots for New Hampshire soil data (Figure S5); SHAP plots using study area background and state-specific background for PFOS and PFOA (Figure S6); PFOA model partial dependence plots (Figure S7); PFOS model partial dependence plots (Figure S8); upper and lower 95% confidence intervals for probability predictions (Figure S9); groundwater detection frequency of PFOA and PFOS by bins of pH and TOC after removing wells within the consent decree (Figure S10); groundwater detection frequency of PFOA and PFOS by bins of pH and TOC after removing wells within the air model boundary (Figure S11); groundwater detection frequency of PFOA and PFOS by bins of pH and TOC after removing wells within 30 km (Figure S12); depth-weighted average pH and organic matter from the POLARIS6 data set by lithogeochemical groups (Figure S13); PFOS and PFOA model probabilities of exceeding the New Hampshire soil remediation standards by lithogeochemical group (Figure S14); groundwater detection frequencies for PFOS and PFOA grouped by lithology without wells within the consent decree boundary (Figure S15); groundwater detection frequencies for PFOS and PFOA grouped by lithology without wells within the air model boundary (Figure S16); groundwater detection frequencies for PFOS and PFOA grouped by lithology without wells within 30 km (Figure S17); method detection limits from the three statewide studies (Table S1); potential predictor variables included in modeling (Table S2); Wilcoxon one-sided signed rank test for differences between the 0–6 in. and 6–12 in. sampling intervals (Table S3); and references (PDF)

## ∥

Weston Solutions, Concord, NH 03301, United States
