# Supplementary material for: Predictions of Anthropogenic Background PFAS Concentrations in Soil and Relation to Bedrock Lithology and Groundwater Quality
Source: Environ Sci Technol. 2026 Jun 1;60(23):16792–804. doi: 10.1021/acs.est.5c16810 (PMC13276898; doi:10.1021/acs.est.5c16810)
Supplement: Supplementary file 1 [file es5c16810_si_001.pdf]

Supporting Information

Predictions of anthropogenic background PFAS concentrations in soil and relation to bedrock lithology and groundwater quality

Andrea K. Tokranov [a], Leah M. Santangelo [a], Joseph D. Ayotte [a], Sydney M. Welch [a], Kate Emma A. Schlosser [b], Jeffrey M. Marts [b], Anthony F. Drouin [b, 1], Harrison Roakes[c]

[a.] U.S. Geological Survey, Pembroke, NH 03275, United States

[b] New Hampshire Department of Environmental Services, Concord, NH 03302, United States

[c.] Sanborn, Head & Associates, Inc., Bedford, NH 03110, United States

[1] Present address: Weston Solutions, Concord, NH 03301, United States

[*] Corresponding author: atokranov@usgs.gov

33 Pages
17 Figures
3 Tables

Any use of trade, firm, or product names is for descriptive purposes only and does not imply endorsement by the U.S. Government.

KEYWORDS. PFAS, soils, boosted regression tree modeling, fate and transport, groundwater, lithology, leaching

SYNOPSIS. Soil PFAS background predictions for northern New England indicate widespread elevated concentrations. Low soil concentrations may be indicative of underlying groundwater vulnerability.

Materials and Methods

There are two freely available data releases associated with this paper. The first contains information about the sampling, analysis, and results from sampling within New Hampshire,[1] while the second contains compiled per- and polyfluoroalkyl substances (PFAS) data from Maine, Vermont, and New Hampshire, model code, training data, and model output[2]. Below is a brief description of the study design, methods, analysis, and quality control for the New Hampshire study.

New Hampshire sampling study design. Soil sampling sites were randomly determined following an equal-area grid approach.[3] The state of New Hampshire was gridded into 100 equal-area grid cells after excluding all land classified by the 2016 National Land Cover Database[4] as developed, agricultural (pasture, hay, cultivated crops), open water, or unclassified. To eliminate potential contamination from nearby sources, a 500-meter buffer was placed around all parcels identified to have known or potential PFAS use or release (e.g., fire training sites, fire stations, wastewater treatment facilities, airports, landfills). Sampling sites were located as close as possible to the random location identified that fit the above criteria. All samples were collected at least 20 meters away from the road with two exceptions (S070, and S029) where significant undergrowth and/or steep drop-offs were present. Both sites were sampled near low-traffic dirt roads greater than 5 m from the roadside.

Soil Sampling. Samples from 0-6 inches depth were collected at all locations. At 50 locations, soil samples were collected from 6-12 inches in depth. At 6 locations, profiles were collected in 6-inch increments to a maximum of 36 inches below the land surface. All depths refer to depth below land surface (depth = 0 inches) after clearing away surface debris such as leaf litter and sticks.

Field personnel wore cotton outer layers while sampling along with PFAS-free boots and cold-weather gear (as needed). Only personal care products marketed as PFAS-free were worn (sunscreen, hand sanitizer, etc.).

Stainless steel equipment was used to collect soil samples. Samples were composited by combining soil from three locations at the extent of a grid approximately 0.6 meter by 0.6 meter in size into a stainless steel bowl and mixing with a stainless steel trowel. Equipment was cleaned between each sample after brushing off loose soil by rinsing with deionized (DI) water, scrubbing with Liquinox® mixed with DI water, rinsing three times with additional DI water, and finally rinsing with liquid chromatography/mass spectrometry (LC-MS) grade water. The DI and LC-MS water was tested for PFAS and subjected to the Total Oxidizable Precursor Assay (TOPA) prior to the start of the study and contained no concentrations above the Method Detection Limit (MDL).

Sample Analyses. All soil samples were analyzed for 36 PFAS compounds, pH, total organic carbon (TOC), and percent moisture. TOPA, which oxidizes perfluoroalkyl acid (PFAA) precursors into PFAA, was completed for 50 locations in the 0-6 inch sampling interval. Autoclaved-citrate extractable protein (hereafter referred to as protein) analysis was conducted for 91 samples in the 0-6 inch sampling interval. All samples were visually classified using the National Soil Survey Center Natural Resources Conservation Service U.S. Department of Agriculture Field Book for Describing and Sampling Soils.[5] PFAS, TOPA, TOC, and moisture were analyzed by Eurofins Lancaster Laboratories Environment Testing, LLC (Eurofins; Lancaster, PA, USA). PFAS and TOPA analyses were completed using liquid chromatography tandem mass spectrometry (LC-MS/MS) and isotope dilution analysis. TOC analysis was conducted using the EPA Lloyd Kahn method[6] (solids) or EPA method 415.1[7] (aqueous), and moisture content was analyzed using standard method 2540. Protein analysis was completed at the

Cornell Soil Health Laboratory[8] and soil pH (calcium chloride) was completed at the U.S. Geological Survey. All results and additional detail on sampling and analysis are publicly available in the associated data release.[1]

Quality Assurance/Quality Control. Throughout the study a total of 22 equipment blanks were collected and analyzed for PFAS, TOPA, and TOC to monitor for contamination introduced by the equipment, field personnel, or atmosphere. Equipment blanks were collected by decanting LC-MS water over the sampling equipment and collecting the water in the stainless steel sampling bowl before distributing the water to sampling containers. Additionally, three LC-MS water blanks and two DI water source solution blanks were collected for PFAS and TOPA, and two LC-MS water blanks and one DI water blank were collected for TOC. Replicates were collected for approximately 10% of the soil samples for all analyses. At the laboratory, matrix spikes (n = 20) were conducted for PFAS to evaluate recovery, in addition to laboratory blanks and laboratory control spikes.

Data Quality Evaluation for New Hampshire. Evaluation of the equipment and source solution blanks indicated no anticipated effect on soil concentrations. However, method blanks from the laboratory resulted in censoring of one sample for perfluorohexanoic acid (PFHxA), 25 samples for perfluorobutane sulfonic acid (PFBS), and 5 samples for 6:2 fluorotelomer sulfonic acid based on the requirement for all sample results to be greater than 5 times the associated method blank concentration. In all cases, the censored results were less than or close to the reporting level. TOPA data were similarly censored. Average relative percent difference for duplicate samples (n = 15) was less than 30%, and average relative standard deviation for triplicate samples (n = 3) was less than 20% across all PFAS compounds, pH, protein, moisture content, and TOC. Matrix spike and matrix spike duplicate recoveries were within ±30% for 92% of the data (Figure S1). Compounds

with values frequently outside this range were perfluorooctane sulfonamide (PFOSA) and hexafluoropropylene oxide dimer acid (HFPO-DA), with frequent over-recoveries, and long chain compounds perfluorooctadecanoic acid (PFODA), perfluorododecane sulfonic acid (PFDoS), and 1H,1H, 2H, 2H-perfluorododecane sulfonic acid (10:2 FtS), which often had low recoveries. These compounds were not frequently detected in our soil samples.

A follow-up confirmatory study of soil sample results in New Hampshire was conducted to ensure sampling was representative of the area.[9] Evaluation of this follow-on work indicated that only 1 of the 15 sites originally sampled would have resulted in a different model classification for perfluorooctanoic acid (PFOA) only (i.e., initial results indicated PFOA was below 0.4 ng/g, but follow-up sampling indicated PFOA exceeded 0.4 ng/g).

Sampling design for Maine and Vermont

The properties sampled during previous studies in Maine[10] and Vermont[11, 12] were generally publicly owned or conserved lands and were screened for nearby, potential PFAS contamination point sources. Unlike the New Hampshire study, which focused sample collection on undeveloped lands, the Maine and Vermont studies included samples from lightly developed properties, such as parks and public greens. Vermont followed a gridded approach to sampling the state, approximately sampled from alternating grid cells, and screened for any potential nearby sources.[12] For Maine, four sampling locations were chosen within each of the 16 counties to ensure geographic coverage, with half of the samples taken in urban settings and the other half in non-urban areas.[10] Sampling locations were ≥500 feet from any potential PFAS source.[10]

Calculation of perfluorooctanoic acid (PFOA) and perfluorooctane sulfonic acid (PFOS) mass residing in Vermont, Maine, and New Hampshire soils

Grid cells where PFOA/PFOS were predicted to be detected at concentrations above the New Hampshire soil remediation threshold value were assigned the value of the threshold: 0.4 nanograms per gram (ng/g) for PFOA and 0.5 ng/g for PFOS. Grid cells where soils were not predicted to exceed threshold concentrations were assigned a value of 0 ng/g. This provides a lower bound estimate on concentration. The concentrations were then multiplied by the soil bulk density values for each grid cell. Mean soil bulk density from the Probabilistic Remapping of SSURGO (POLARIS; SSURGO = Soil Survey Geographic Database) dataset for the 0-5 centimeter (cm) and 5-15 cm intervals were downloaded,[13] and the average depth-weighted value of bulk density within the full 0-15 cm interval (~6 inches) was calculated for each raster cell. The resulting bulk density file was then resampled to the 1x1 square kilometer grid cells used for this study and multiplied by the modeled PFOA or PFOS predictions. Total mass for each grid cell was then calculated using the volume in a 1x1 square kilometer grid cell with 6 inch soil depth, and the grid cell values within each state were summed.

The equivalent volume in terms of years of annual groundwater recharge was calculated using the total land area for the study (129,720 km^2) and assuming an average annual normalized groundwater recharge rate of 21.0 inches per year[14].

Analysis of the effect of a manufacturing facility with known PFOA emissions

The presence of a manufacturing facility with a known history of PFOA emissions south of Manchester, New Hampshire may complicate analysis.[15] Because it is unknown exactly what the impact extent is from this manufacturing facility, we removed wells from our analysis that correspond to three boundary scenarios (listed smallest to largest): (1) within the area covered by

the consent decree boundary established between the New Hampshire Department of Environmental Services and the manufacturer, which includes areas where investigation and remediation are ongoing,[16] (2) within the area covered by the model domain for an atmospheric deposition model for the facility,[17] and (3) within a 30 kilometer radius around the facility.

Lithogeochemical groupings

Lithogeochemical groupings were modified from previous work.[18, 19] Specifically, "Peraluminous Granite", "Granite, other", "Alkali Granite", "Felsic Volcanics", and "Grenville Granite" were grouped into the "Felsic Igneous" category. "Mafic rocks", "Ultramafic rocks", and "Basalt" were grouped into the "Mafic Igneous" category. "Sulfidic Schists" remained in a single category. "Carbonate Rocks", "Calcpelite", and "Calcgranofels" were grouped into a "Calcareous Rocks" category. "Pelitic Rocks" and "Metamorphic Rocks, other" were further modified by whether they were Undifferentiated or Calcareous.

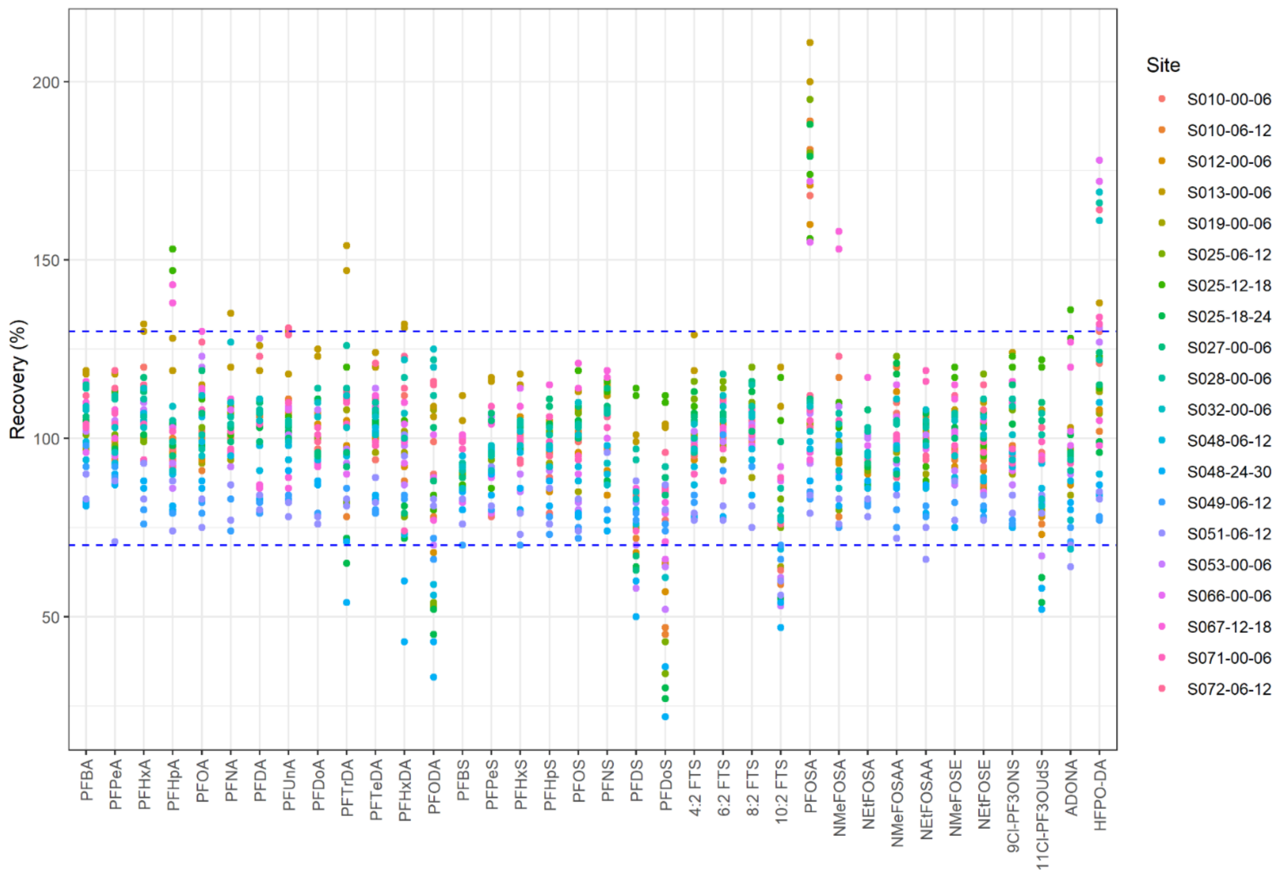

Figure S1. Recoveries of matrix spike (MS) and matrix spike duplicates (MSD) run on New Hampshire soils samples throughout the study.[1] Blue dashed lines are set at 70% and 130% as a visual aid. See Table S1 for majority of compound abbreviations. PFDoS = perfluorododecanesulfonic acid; 4:2 FTS = 1H,1H, 2H, 2H-perfluorohexane sulfonic acid; 6:2 FTS = 1H,1H, 2H, 2H-perfluorooctane sulfonic acid; 8:2 FTS = 1H,1H, 2H, 2H-perfluorodecane sulfonic acid; 10:2 FTS = 1H,1H, 2H, 2H-perfluorododecane sulfonic acid; PFOSA = perfluorooctanesulfonamide; NMeFOSA = N-methyl perfluorooctanesulfonamide; NEtFOSA = N-ethyl perfluorooctanesulfonamide; NMeFOSAA = N-methyl perfluorooctanesulfonamidoacetic acid; NEtFOSAA = N-ethyl perfluorooctanesulfonamidoacetic acid; NMeFOSE = N-methyl perfluorooctanesulfonamidoethanol; NEtFOSE = N-ethyl perfluorooctanesulfonamidoethanol; 9Cl-PF3ONS = 9-chlorohexadecafluoro-3-oxanonane-1-sulfonic acid; 11Cl-PF3OUdS = 11-chloroeicosafluoro-3-oxaundecane-1-sulfonic acid; ADONA = 4,8-dioxa-3H-perfluorononanoic acid; HFPO-DA = hexafluoropropylene oxide dimer acid.

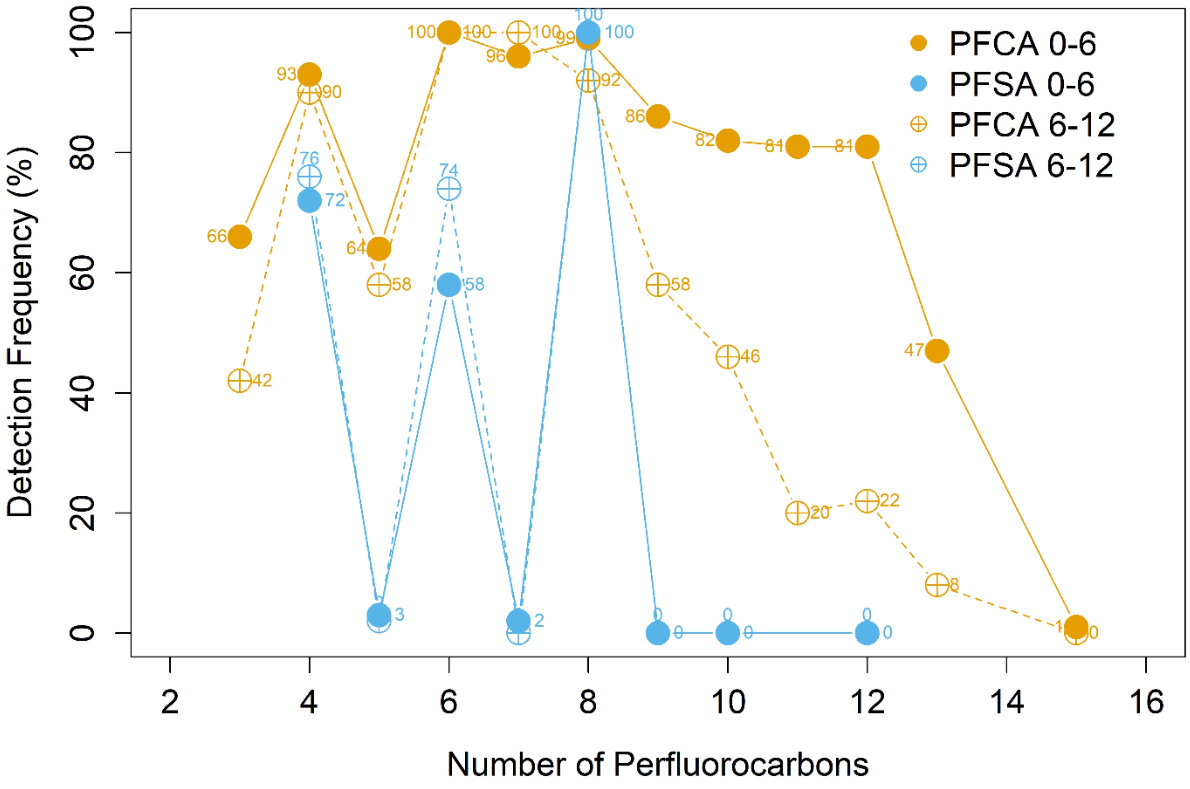

Figure S2. Detection frequency (%) of perfluoroalkyl carboxylic acid (PFCA) and perfluoroalkane sulfonic acid (PFSA) compounds from 0-6 inch (solid circles) and 6-12 inch (open circles) depths by perfluorocarbon chain length.[1] Note that the number of perfluorinated carbons is on the x-axis (i.e., perfluorooctanoic acid, PFOA, is plotted at 7).

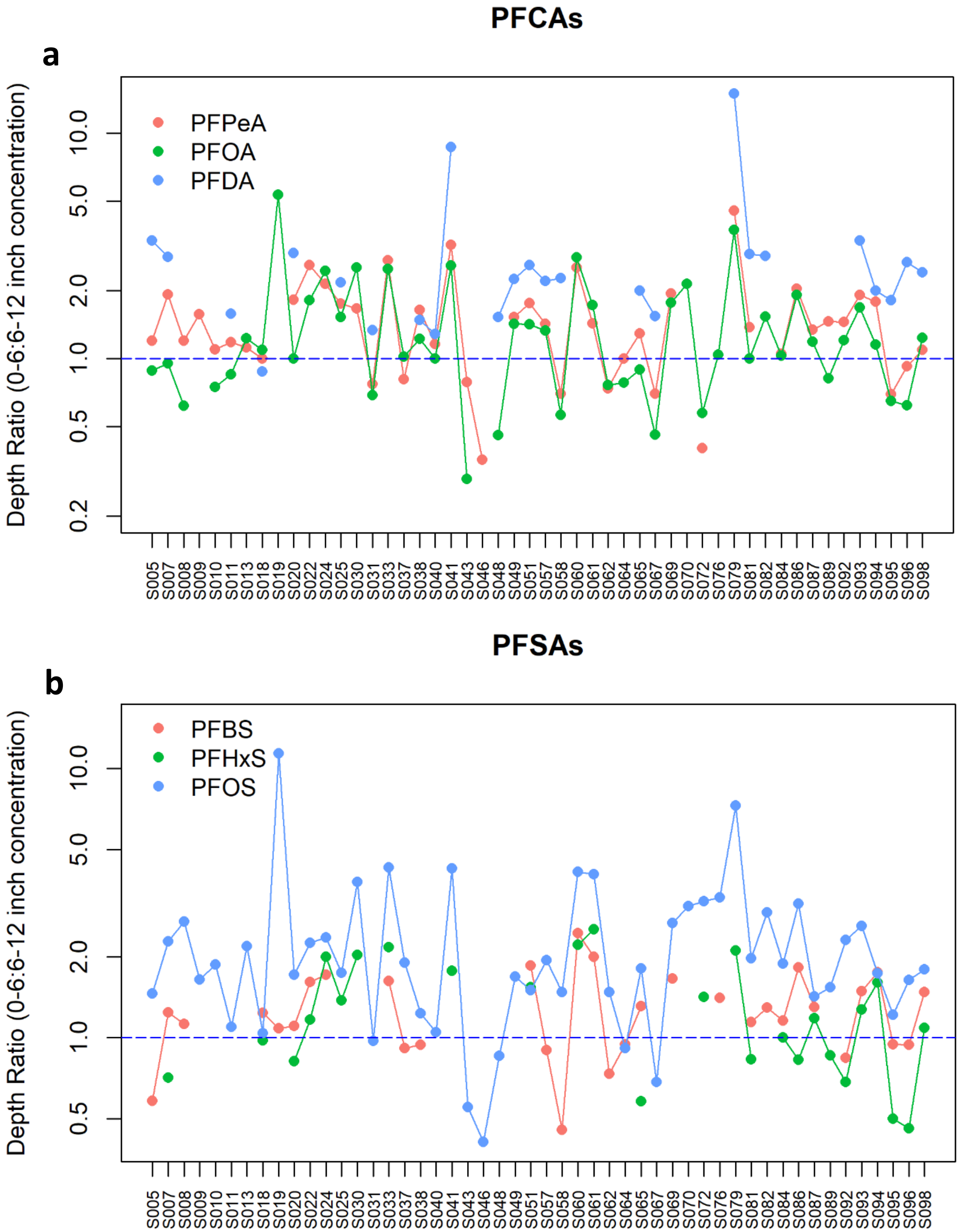

Figure S3. The ratio of the PFAS concentration in the 0-6 inch sample to the 6-12 inch sample for select (a) perfluoroalkyl carboxylic acids (PFCAs), and (b) perfluoroalkane sulfonic acids (PFSAs). Missing values are where there were non-detects in at least one of the depth intervals. The dashed blue lines indicate a ratio of 1 (no concentration difference between the top 0-6 inch and bottom 6-12 inch sample). Abbreviated site names are shown on the x axis. PFPeA = perfluoropentanoic acid; PFOA = perfluorooctanoic acid; PFDA = perfluorodecanoic acid; PFBS = perfluorobutane sulfonic acid; PFHxS = perfluorohexane sulfonic acid (PFHxS); PFOS = perfluorooctane sulfonic acid.

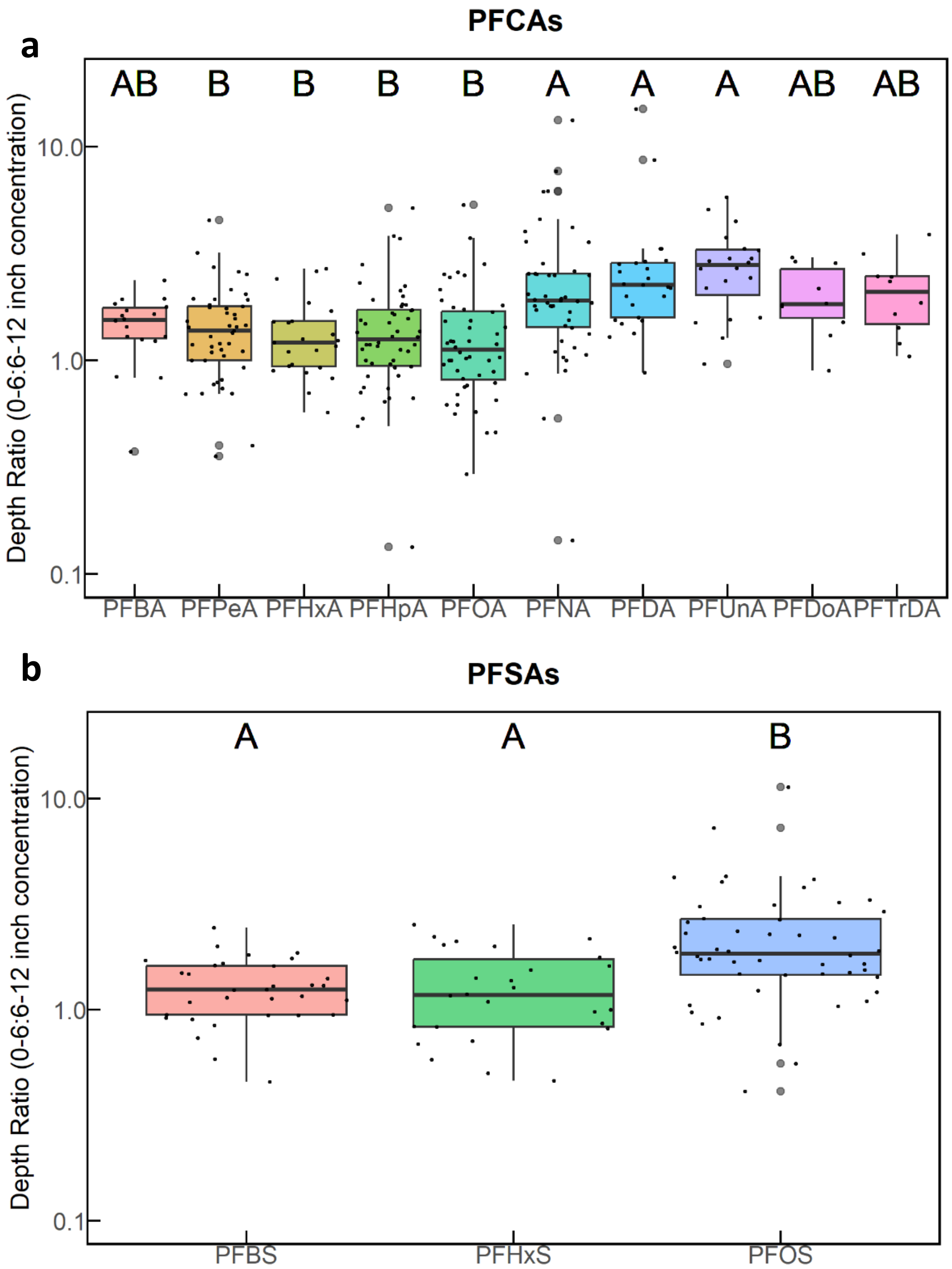

Figure S4. The ratio of the PFAS concentration in the 0-6 inch sample to the 6-12 inch sample by compound for (a) perfluoroalkyl carboxylic acids (PFCAs), and (b) perfluoroalkane sulfonic acids (PFSAs). Different letters above each boxplot represent a significant difference from the implementation of a Kruskal-Wallis and *post hoc* Dunn's test (p<0.05). PFBA = perfluorobutanoic acid; PFPeA = perfluoropentanoic acid; PFHxA = perfluorohexanoic acid; PFHpA = perfluoroheptanoic acid; PFOA = perfluorooctanoic acid; PFNA = perfluorononanoic acid; PFDA = perfluorodecanoic acid; PFUnA = perfluoroundecanoic acid; PFDoA = perfluorododecanoic acid; PFTrDA = perfluorotridecanoic acid; PFBS = perfluorobutane sulfonic acid; PFHxS = perfluorohexane sulfonic acid (PFHxS); PFOS = perfluorooctane sulfonic acid.

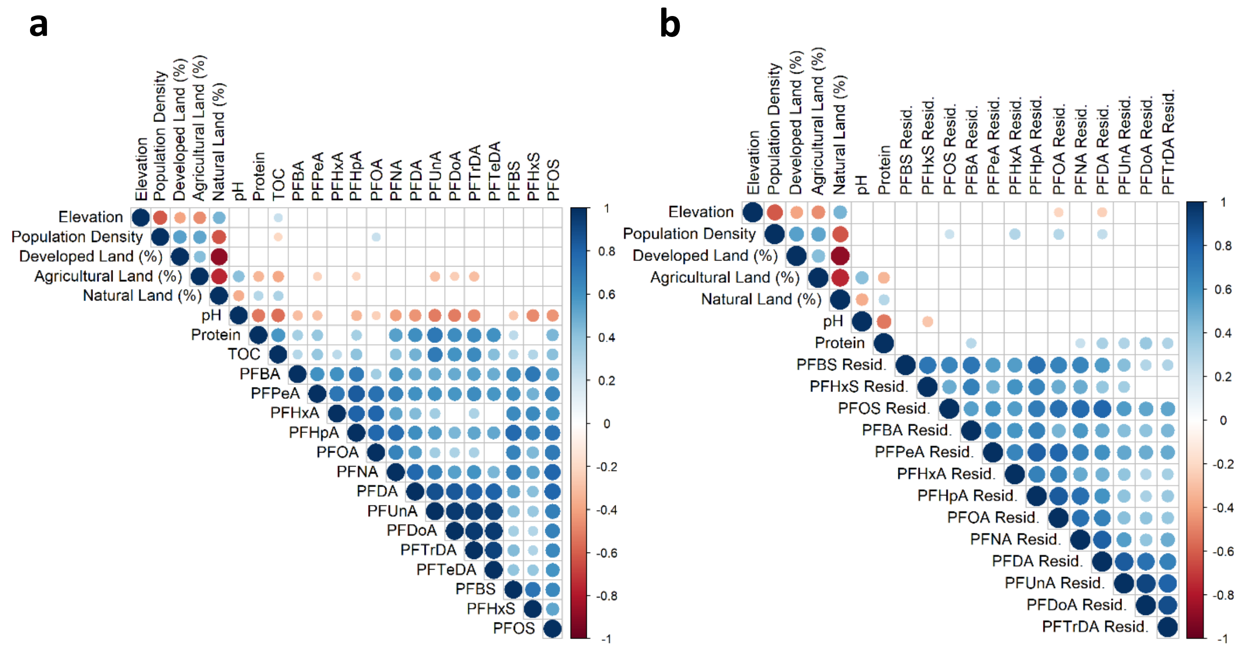

Figure S5. Spearman correlation plots for all samples from the 0-6 inch sampling interval (n = 100) using (a) unmodified PFAS concentrations, and (b) PFAS residuals after regressing each PFAS compound on TOC using all available primary data from 0-36 inches in depth. Spearman correlation coefficients are indicated by the red and blue scale. Non-significant (p-value > 0.05) results are not shown. Developed, agricultural, and natural land use was calculated using a 1-kilometer buffer around the sample points. See Table S2 for land use, elevation, and population density references. PFBA = perfluorobutanoic acid; PFPeA = perfluoropentanoic acid; PFHxA = perfluorohexanoic acid; PFHpA = perfluoroheptanoic acid; PFOA = perfluorooctanoic acid; PFNA = perfluorononanoic acid; PFDA = perfluorodecanoic acid; PFUnA = perfluoroundecanoic acid; PFDoA = perfluorododecanoic acid; PFTrDA = perfluorotridecanoic acid; PFBS = perfluorobutane sulfonic acid; PFHxS = perfluorohexane sulfonic acid; PFOS = perfluorooctane sulfonic acid; TOC = total organic carbon.

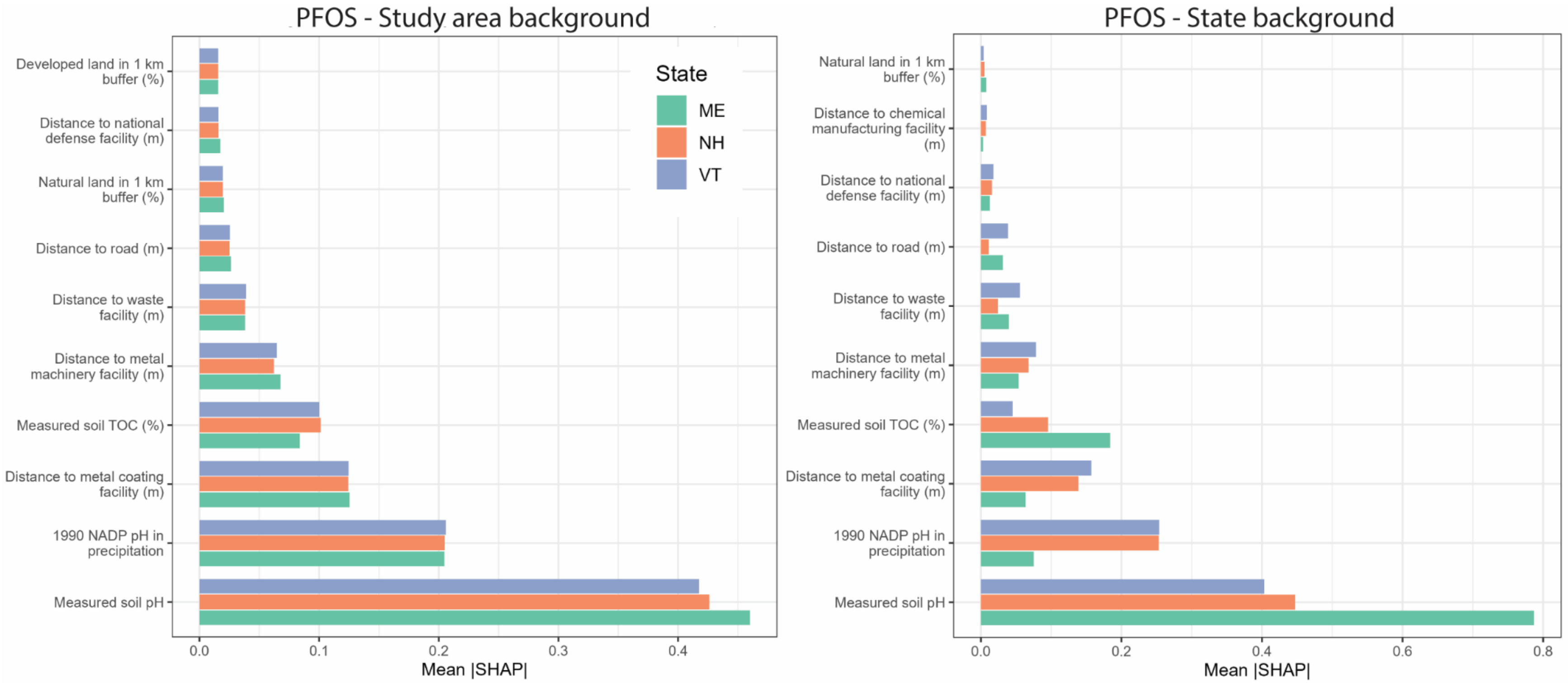

Figure S6a. SHapley Additive exPlanation (SHAP) values for perfluorooctane sulfonic acid (PFOS) using either the study area background or state-specific background distribution for Vermont, Maine, and New Hampshire. All SHAP values for Vermont were calculated using the study area background values for soil pH due to the absence of in-state pH measurements. TOC = Total organic carbon; NADP = National Atmospheric Deposition Program; m = meter; km = kilometer.

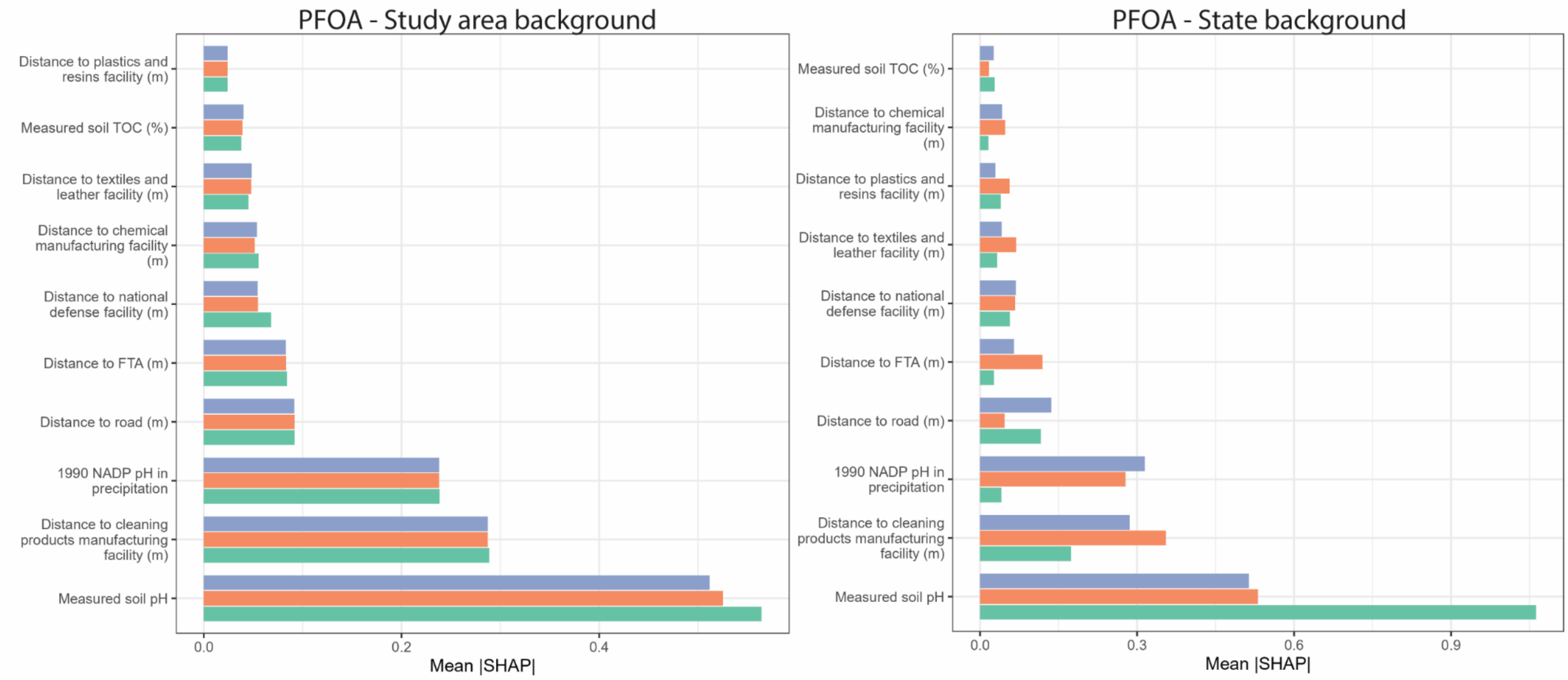

Figure S6b. SHapley Additive exPlanation (SHAP) values for perfluorooctanoic acid (PFOA) using either the study area background or state-specific background distribution for Vermont, Maine, and New Hampshire. All SHAP values for Vermont were calculated using the study area background values for soil pH due to the absence of in-state pH measurements. TOC = Total organic carbon; NADP = National Atmospheric Deposition Program; FTA = Fire training area; m = meter.

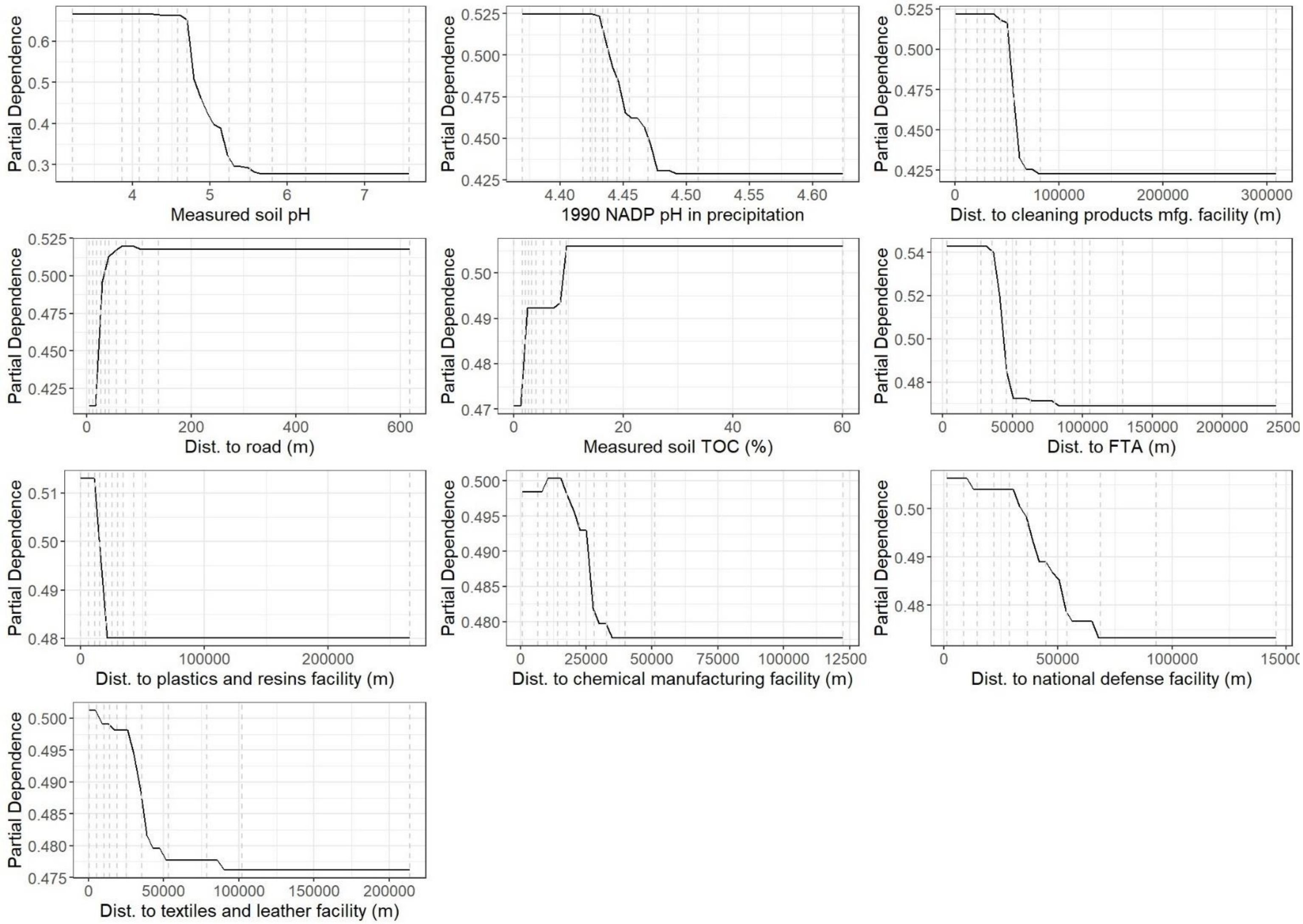

Figure S7. Partial dependence plots for each variable (Table S2) in the perfluorooctanoic acid (PFOA) model. Vertical grey dashed lines indicate deciles of the training data, including minimum and maximum values. Note the y-axis ranges vary between plots. TOC = Total organic carbon; NADP = National Atmospheric Deposition Program; FTA = Fire training area; m = meter; Dist. = distance, mfg = manufacturing.

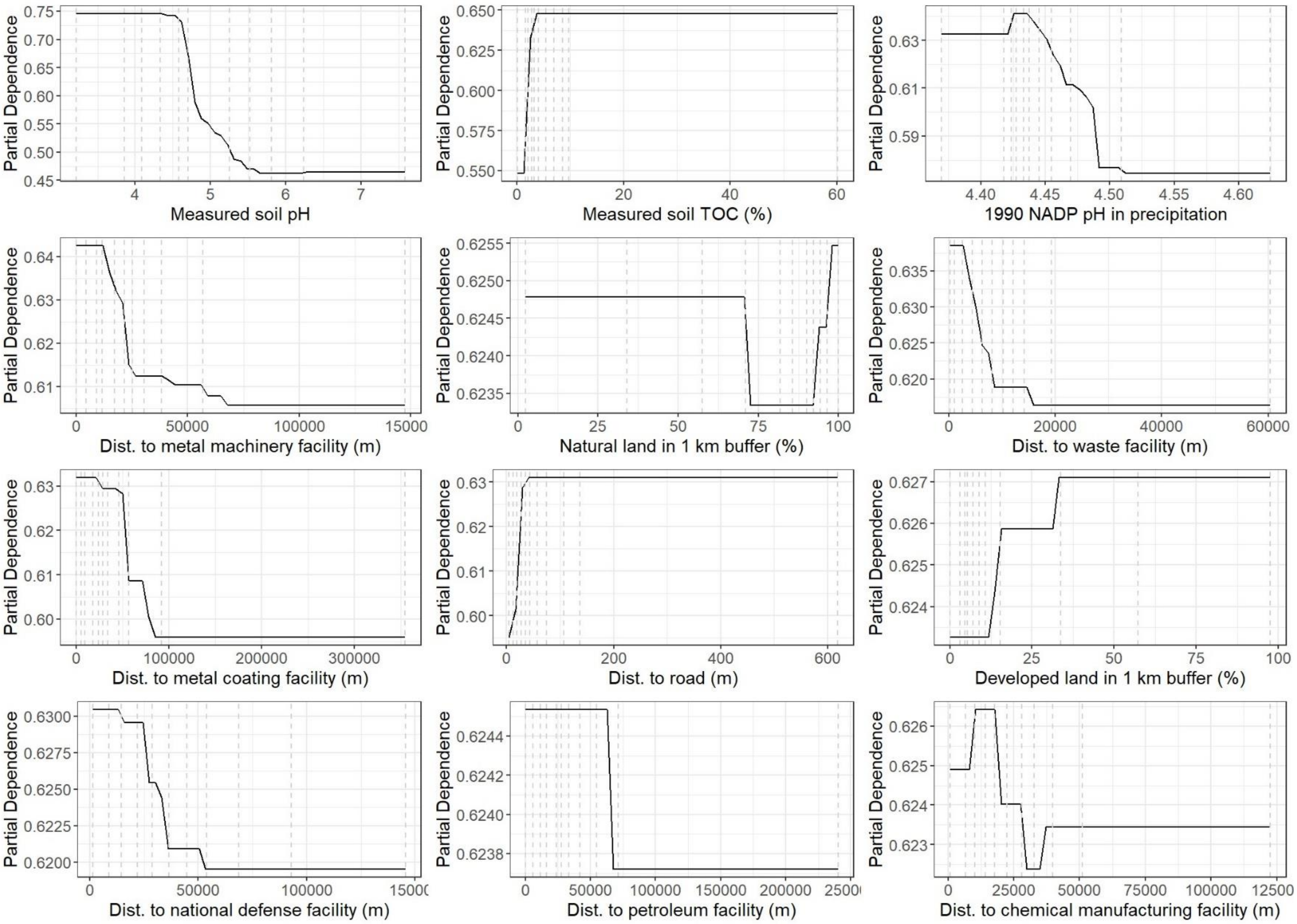

Figure S8. Partial dependence plots for each variable (Table S2) in the perfluorooctane sulfonic acid (PFOS) model. Vertical grey dashed lines indicate deciles of the training data, including minimum and maximum values. Note the y-axis ranges vary between plots. TOC = Total organic carbon; NADP = National Atmospheric Deposition Program; km = kilometer; m = meter; Dist. = distance.

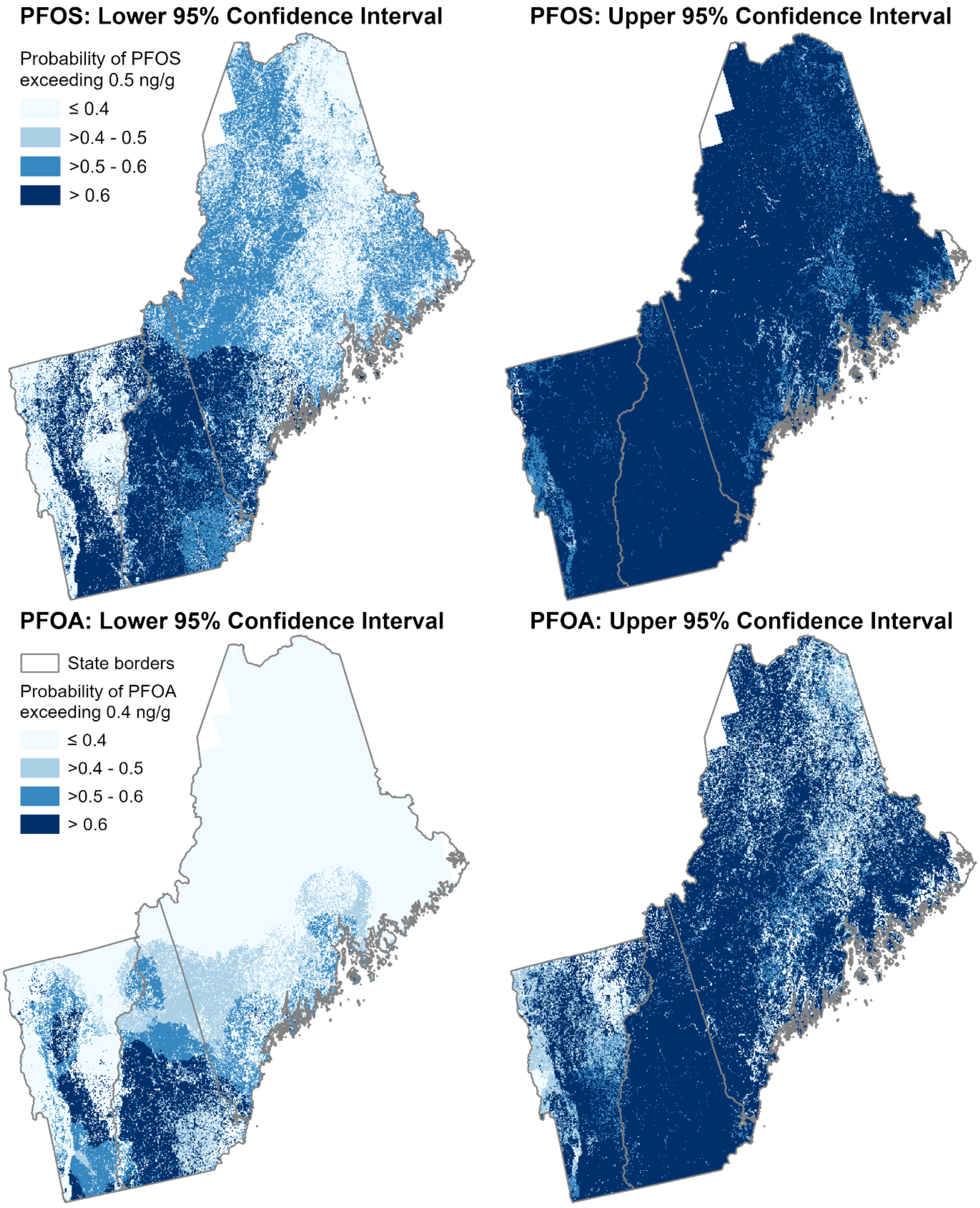

Figure S9. Prediction maps for the lower and upper 95% confidence intervals produced by bootstrapping the training data. Model probabilities of perfluorooctane sulfonic acid (PFOS) and perfluorooctanoic acid (PFOA) concentrations exceeding the New Hampshire Soil Remediation Standards (SRS) of 0.5 ng/g and 0.4 ng/g in soil, respectively.

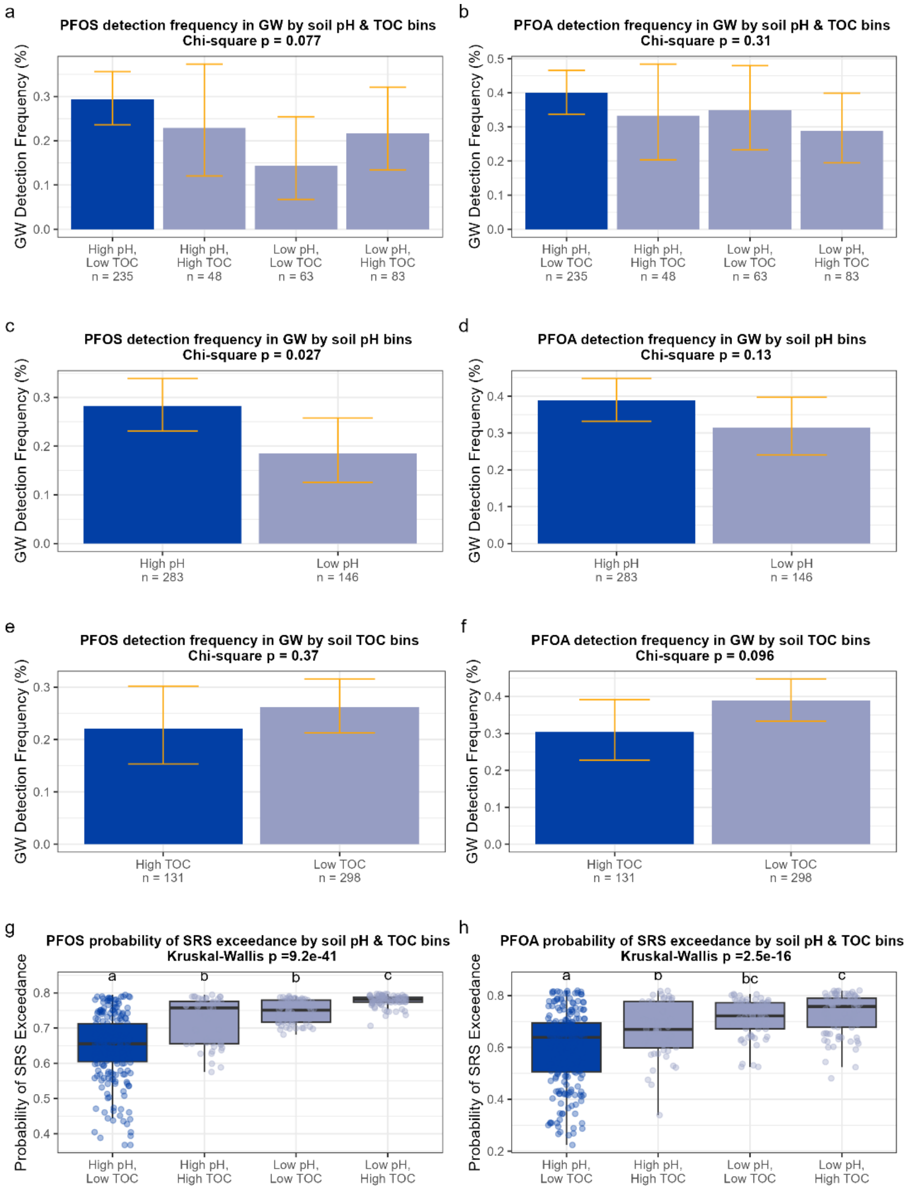

Figure S10. Wells[20] within the consent decree boundary for a manufacturing facility[16] in southern New Hampshire were removed from analysis. **(a-f)** Groundwater (GW) detection frequency of perfluorooctanoic acid (PFOA) and perfluorooctane sulfonic acid (PFOS) by bins of low (<4.46) and high (≥4.46) soil pH and low (<4.76) and high (≥4.76) soil total organic carbon (TOC)[13]. The number of samples in each category are reported as n values on the x-axis. Chi squared *p* values are reported for each plot. Confidence intervals are shown in orange. **(g-h)** Boxplots of probability of exceeding the New Hampshire Soil Remediation Standards (SRS) by bins of low and high pH and TOC using the same classification as panels a-b. SRS values as well as soil pH and soil TOC values are from the grid cell associated with each groundwater well for direct comparison with panels a-f. Kruskal-Wallis *p* values are reported in the plot title, and post-hoc Dunn's test results are reported as letters above the box plots. Common letters indicate no significant difference at $p<0.05$. Boxes extend to first and third quartiles, whiskers extend to 1.5 times the inter-quartile range. Data are overprinted on the plot.

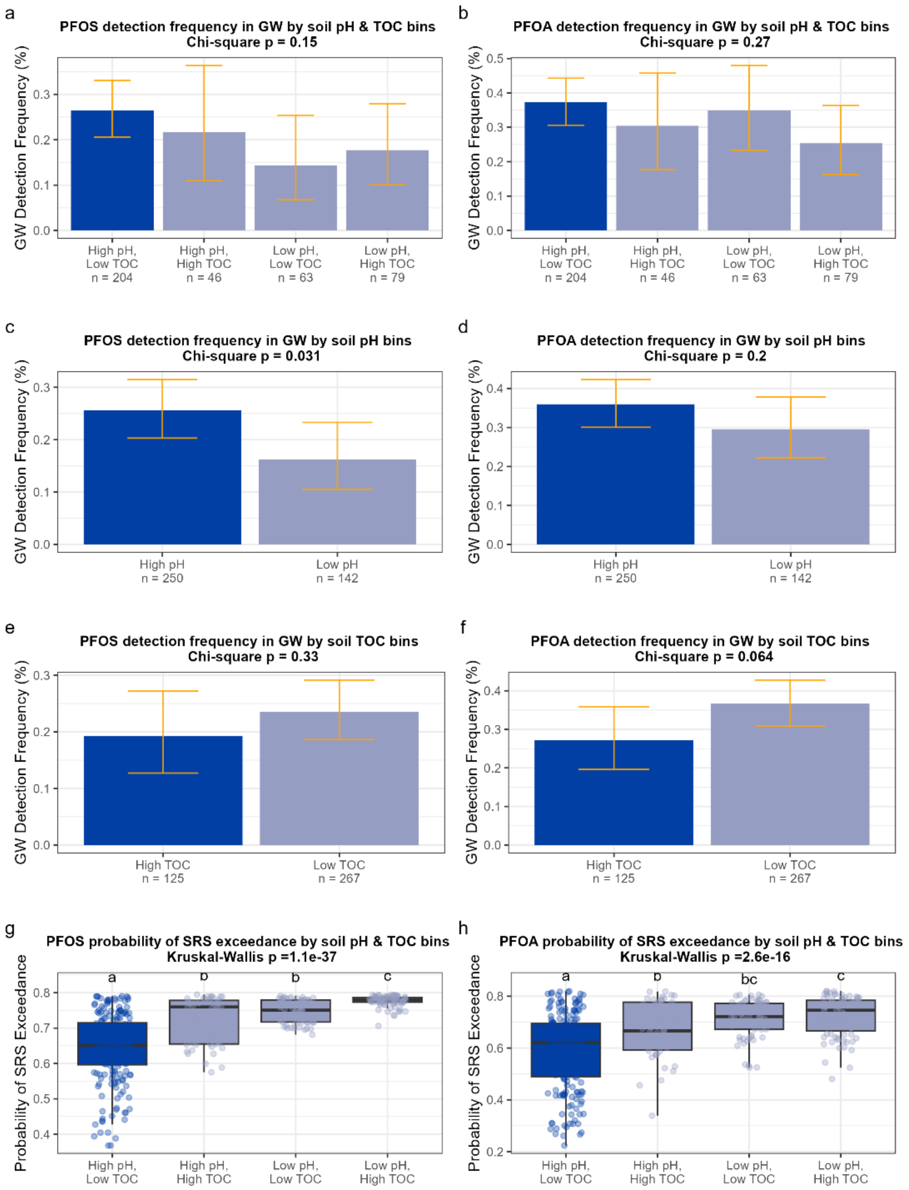

Figure S11. Wells[20] within the model domain for an air deposition model[17] for a manufacturing facility in southern New Hampshire were removed from analysis. **(a-f)** Groundwater (GW) detection frequency of perfluorooctanoic acid (PFOA) and perfluorooctane sulfonic acid (PFOS) by bins of low (<4.46) and high (≥4.46) soil pH and low (<4.76) and high (≥4.76) soil total organic carbon (TOC)[13]. The number of samples in each category are reported as n values on the x-axis. Chi squared *p* values are reported for each plot. Confidence intervals are shown in orange. **(g-h)** Boxplots of probability of exceeding the New Hampshire Soil Remediation Standards (SRS) by bins of low and high pH and TOC using the same classification as panels a-b. SRS values as well as soil pH and soil TOC values are from the grid cell associated with each groundwater well for direct comparison with panels a-f. Kruskal-Wallis *p* values are reported in the plot title, and post-hoc Dunn's test results are reported as letters above the box plots. Common letters indicate no significant difference at $p<0.05$. Boxes extend to first and third quartiles, whiskers extend to 1.5 times the inter-quartile range. Data are overprinted on the plot.

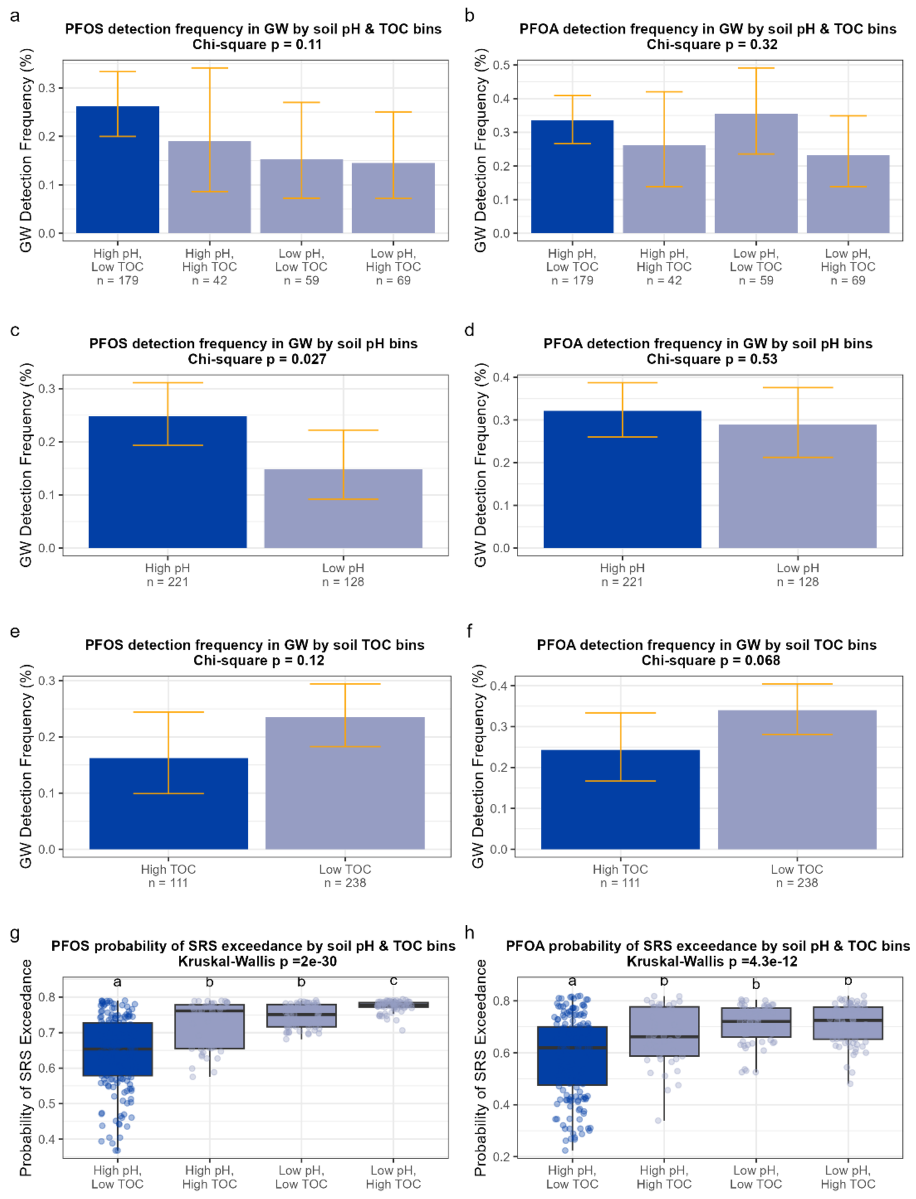

Figure S12. Wells[20] within a 30-kilometer radius of a manufacturing facility in southern New Hampshire were removed from analysis. **(a-f)** Groundwater (GW) detection frequency of perfluorooctanoic acid (PFOA) and perfluorooctane sulfonic acid (PFOS) by bins of low (<4.46) and high (≥4.46) soil pH and low (<4.76) and high (≥4.76) soil total organic carbon (TOC)[13]. The number of samples in each category are reported as n values on the x-axis. Chi squared *p* values are reported for each plot. Confidence intervals are shown in orange. **(g-h)** Boxplots of probability of exceeding the New Hampshire Soil Remediation Standards (SRS) by bins of low and high pH and TOC using the same classification as panels a-b. SRS values as well as soil pH and soil TOC values are from the grid cell associated with each groundwater well for direct comparison with panels a-f. Kruskal-Wallis *p* values are reported in the plot title, and post-hoc Dunn's test results are reported as letters above the box plots. Common letters indicate no significant difference at $p<0.05$. Boxes extend to first and third quartiles, whiskers extend to 1.5 times the inter-quartile range. Data are overprinted on the plot.

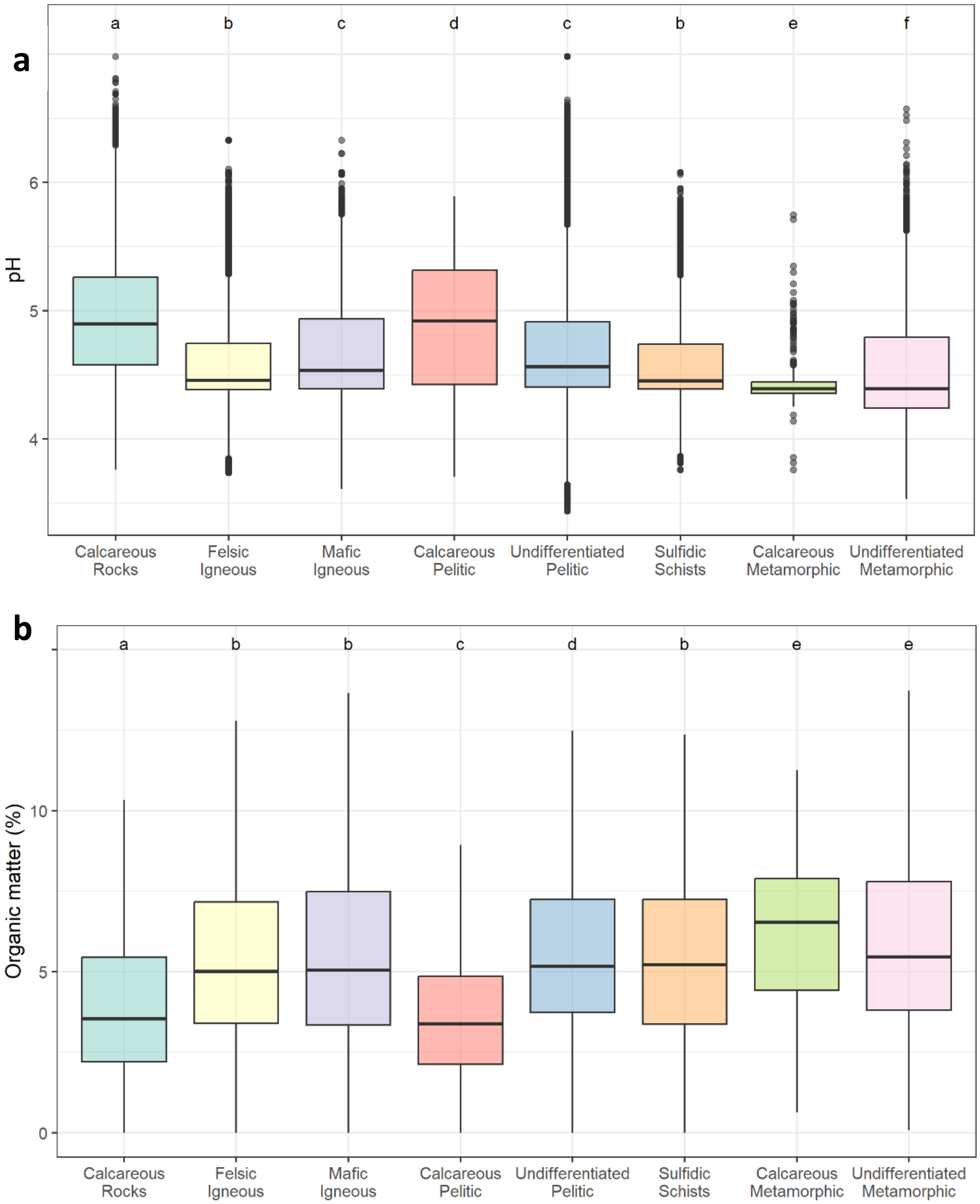

Figure S13. Depth weighted average pH (a) and percent organic matter (b) in the 0-15 cm depth interval from the POLARIS[13] dataset by lithogeochemical groups[18, 19]. The Berwick Formation is in the Calcareous Rocks group. Boxes extend to first and third quartiles, whiskers extend to 1.5 times the inter-quartile range. Outliers are plotted as points beyond whiskers. Outliers were suppressed in the bottom organic matter figure to better see the boxes. A Kruskal-Wallis with post-host Dunn's test and Bonferroni correction was performed and significant differences are denoted by non-overlapping letters.

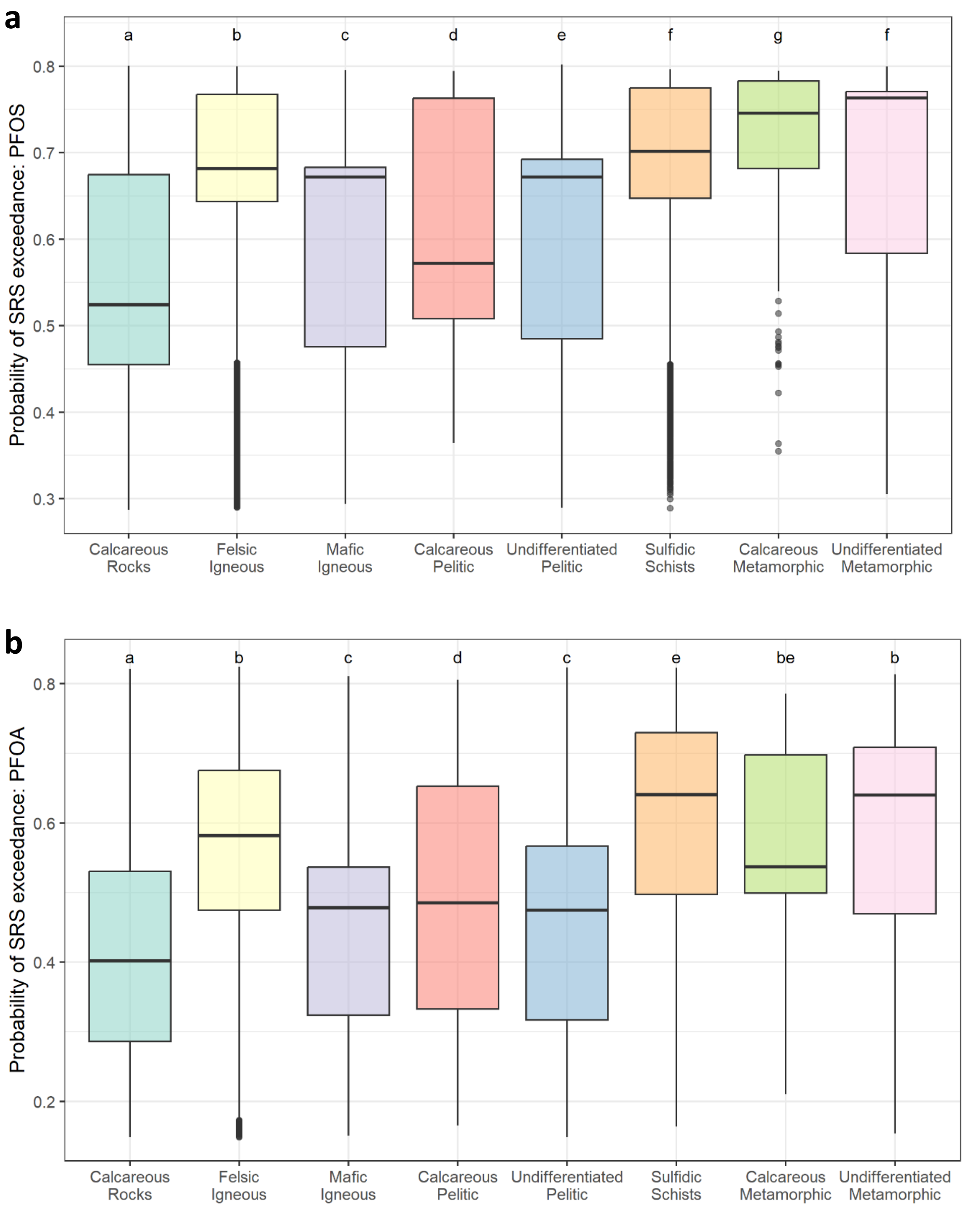

Figure S14. (a) Perfluorooctane sulfonic acid (PFOS) and (b) perfluorooctanoic acid (PFOA) model probabilities of exceeding the New Hampshire soil remediation standard (SRS) by lithogeochemical group[18, 19]. Boxes extend to first and third quartiles, whiskers extend to 1.5 times the inter-quartile range. Outliers are plotted as points beyond whiskers. The Berwick Formation is in the Calcareous Rocks group. A Kruskal-Wallis with post-host Dunn's test and Bonferroni correction was performed and significant differences are denoted by non-overlapping letters.

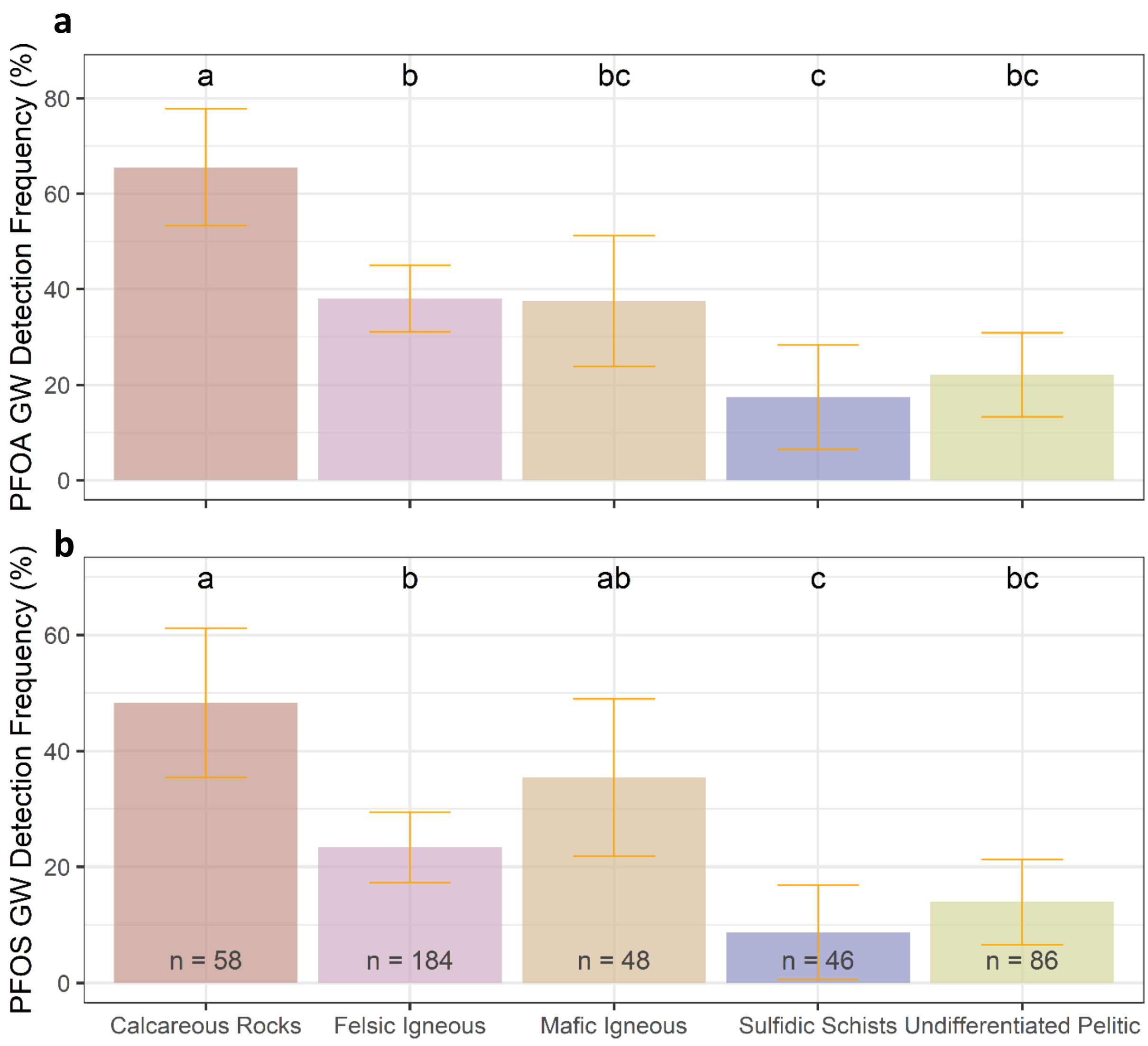

Figure S15. Groundwater[20] (GW) detection frequencies for (a) perfluorooctanoic acid (PFOA) and (b) perfluorooctane sulfonic acid (PFOS) grouped by lithology[18, 19]. Wells within the consent decree boundary for a manufacturing facility[16] in southern New Hampshire were removed from analysis. Orange bars represent confidence intervals. The results for "Undifferentiated Metamorphic" were removed because there were only 9 samples within that category. The Berwick Formation is in the Calcareous Rocks group. Pairwise proportion tests were used to identify significant differences ($p < 0.05$) and are denoted by different letters at the top of each bar.

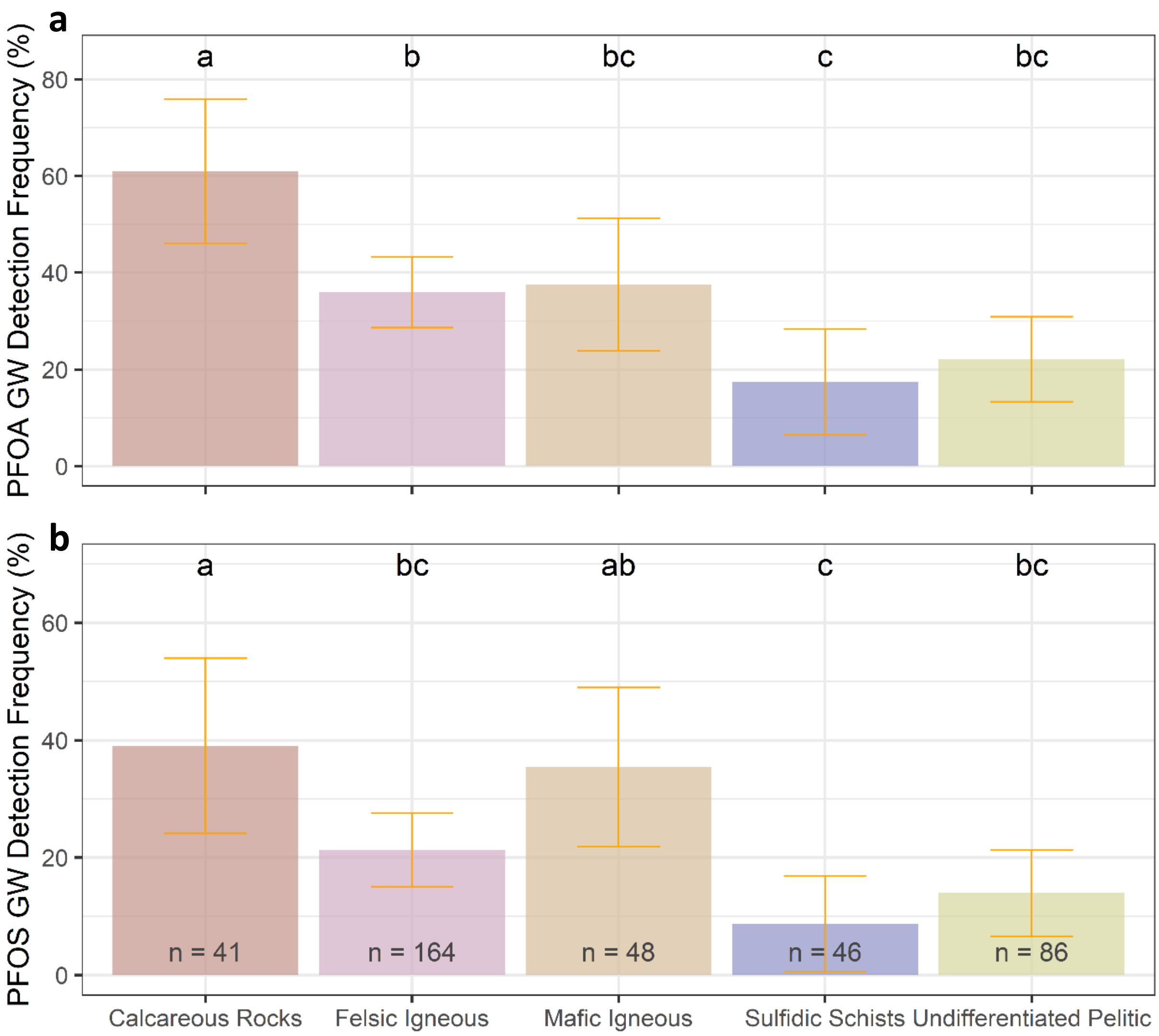

Figure S16. Groundwater[20] (GW) detection frequencies for (a) perfluorooctanoic acid (PFOA) and (b) perfluorooctane sulfonic acid (PFOS) grouped by lithology[18, 19]. Wells within the model domain for an air deposition model[17] for a manufacturing facility in southern New Hampshire were removed from analysis. Orange bars represent confidence intervals. The results for "Undifferentiated Metamorphic" were removed because there were only 9 samples within that category. The Berwick Formation is in the Calcareous Rocks group. Pairwise proportion tests were used to identify significant differences ($p < 0.05$) and are denoted by different letters at the top of each bar.

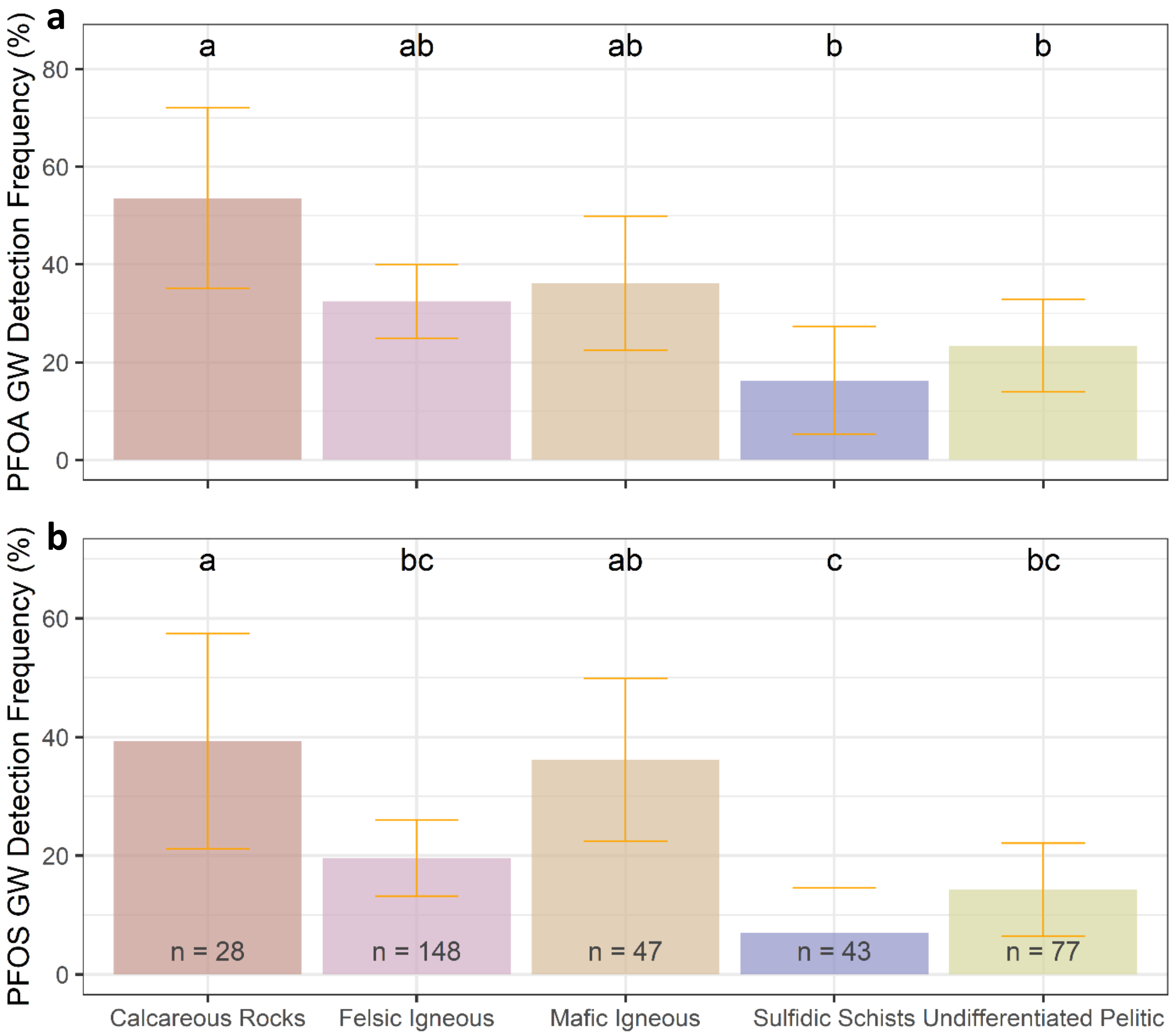

Figure S17. Groundwater[20] (GW) detection frequencies for (a) perfluorooctanoic acid (PFOA) and (b) perfluorooctane sulfonic acid (PFOS) grouped by lithology[18, 19]. Wells within a 30-kilometer radius of a manufacturing facility in southern New Hampshire were removed from analysis. Orange bars represent confidence intervals. The results for "Undifferentiated Metamorphic" were removed because there were only 9 samples within that category. The Berwick Formation is in the Calcareous Rocks group. Pairwise proportion tests were used to identify significant differences ($p < 0.05$) and are denoted by different letters at the top of each bar.

Table S1. Method detection limits (MDLs) from the three statewide studies.[1, 10-12] Both the median and maximum MDLs were provided for the Maine (ME) and New Hampshire (NH) studies, as MDLs varied by sample and compound. VT = Vermont.

Analyte abbreviation	Analyte name	VT MDL	ME median MDL	ME maximum MDL	NH median MDL (for 100 primary 0-6 inch samples)	NH maximum MDL (for 100 primary 0-6 inch samples)
PFBA	Perfluorobutanoic acid	0.1	0.027	0.083	0.36	1.9
PFPeA	Perfluoropentanoic acid	0.07	0.054	0.168	0.06	0.31
PFHxA	Perfluorohexanoic acid	0.0076	0.062	0.192	0.15	0.77
PFHpA	Perfluoroheptanoic acid	0.0044	0.053	0.165	0.03	0.15
PFOA	Perfluorooctanoic acid	0.007	0.05	0.153	0.06	0.31
PFNA	Perfluorononanoic acid	0.0097	0.089	0.274	0.03	0.15
PFDA	Perfluorodecanoic acid	0.008	0.079	0.245	0.03	0.15
PFUnA	Perfluoroundecanoic acid	0.007	0.055	0.171	0.03	0.15
PFDoA	Perfluorododecanoic acid	0.011	0.083	0.256	0.03	0.15
PFTrDA	Perfluorotridecanoic acid	0.013	0.242	0.748	0.03	0.15
PFTeDA	Perfluorotetradecanoic acid	0.021	0.064	0.198	0.03	0.15
PFHxDA	Perfluorohexadecanoic acid	0.023	0.142	0.439	0.06	0.31
PFODA	Perfluorooctadecanoic acid	0.024	0.202	0.626		0.15
PFBS	Perfluorobutane sulfonic acid	0.006	0.046	0.143	0.03	0.15
PFPeS	Perfluoropentane sulfonic acid		0.099	0.306	0.03	0.15
PFHxS	Perfluorohexane sulfonic acid	0.014	0.072	0.221	0.03	0.15
PFHpS	Perfluoroheptane sulfonic acid		0.161	0.499	0.03	0.15
PFOS	Perfluorooctane sulfonic acid	0.005	0.154	0.476	0.06	0.31
PFNS	Perfluorononane sulfonic acid		0.353	1.09	0.03	0.15
PFDS	Perfluorodecane sulfonic acid	0.0053	0.181	0.56	0.03	0.15

Table S2. Potential predictor variables included in modeling. Note that measured pH and TOC were used for model training and pH and organic matter from POLARIS were used for predictions.

Variable	Unit	Description	Reference	Notes
pH	unitless	Measured soil pH	[1, 10]	Used for model training only. Soil pH was adjusted for New Hampshire data from calcium chloride pH to water pH using equations from Miller and Kissel, 2010[21] (Figure 1)
TOC	percent	Measured soil Total Organic Carbon (TOC)	[1, 10-12]	Used for model training only.
Organic carbon POLARIS	percent	Depth-weighted mean percentage of soil organic carbon in the 0-15 centimeter depth interval from the Probabilistic Remapping of SSURGO (POLARIS) dataset. SSURGO = Soil Survey Geographic Database	[13]	Organic matter was converted to TOC using a factor of 1.9[22]
pH POLARIS	unitless	Depth-weighted mean pH in the 0-15 centimeter depth interval from the Probabilistic Remapping of SSURGO (POLARIS) dataset. SSURGO = Soil Survey Geographic Database	[13]	
Ksat POLARIS	centimeters per hour	Depth-weighted mean saturated hydraulic conductivity (Ksat) in the 0-15 centimeter depth interval from the Probabilistic Remapping of SSURGO (POLARIS) dataset. SSURGO = Soil Survey Geographic Database	[13]	
silt POLARIS	percent	Depth-weighted mean percentage of soil silt in the 0-15 centimeter depth interval from the Probabilistic Remapping of SSURGO (POLARIS) dataset. SSURGO = Soil Survey Geographic Database	[13]	
Sand POLARIS	percent	Depth-weighted mean percentage of soil sand in the 0-15 centimeter depth interval from the Probabilistic Remapping of SSURGO (POLARIS) dataset. SSURGO = Soil Survey Geographic Database	[13]	
Clay POLARIS	percent	Depth-weighted mean percentage of soil clay in the 0-15 centimeter depth interval from the Probabilistic Remapping of SSURGO (POLARIS) dataset. SSURGO = Soil Survey Geographic Database	[13]	

1990 NADP pH	unitless	National Atmospheric Deposition Program (NADP) gradient map of precipitation-weighted mean pH value from 1990	23	
Natural land	percent	Percentage of natural land within a 1-km buffer around each sampling point	4	
Developed land	percent	Percentage of developed land within a 1-km buffer around each sampling point	4	
Agricultural land	percent	Percentage of agricultural land within a 1-km buffer around each sampling point	4	
Metal coating	meters	Distance to the nearest metal coating facility	24	Distance from closest point to each raster centroid calculated for prediction
Waste	meters	Distance to the nearest waste management facility	24	Distance from closest point to each raster centroid calculated for prediction
Machine metal	meters	Distance to the nearest machine metal facility	24	Distance from closest point to each raster centroid calculated for prediction
Petroleum	meters	Distance to the nearest petroleum facility	24	Distance from closest point to each raster centroid calculated for prediction
Electronics	meters	Distance to the nearest electronics facility	24	Distance from closest point to each raster centroid calculated for prediction
National defense	meters	Distance to the nearest national defense facility	24	Distance from closest point to each raster centroid calculated for prediction
Textiles	meters	Distance to the nearest textiles facility	24	Distance from closest point to each raster centroid calculated for prediction
Paper mills	meters	Distance to the nearest paper mills facility	24	Distance from closest point to each raster centroid calculated for prediction
Cleaning products	meters	Distance to the nearest cleaning products facility	24	Distance from closest point to each raster centroid calculated for prediction

Chemical manufacturing	meters	Distance to the nearest chemical manufacturing facility	24	Distance from closest point to each raster centroid calculated for prediction
Printing	meters	Distance to the nearest printing facility	24	Distance from closest point to each raster centroid calculated for prediction
Paints coatings	meters	Distance to the nearest paints, coating facility	24	Distance from closest point to each raster centroid calculated for prediction
Plastic	meters	Distance to the nearest plastics facility	24	Distance from closest point to each raster centroid calculated for prediction
FTA	meters	Distance to the nearest fire training area (FTA) facility	24	Distance from closest point to each raster centroid calculated for prediction
Airport	meters	Distance to the nearest airport facility	24	Distance from closest point to each raster centroid calculated for prediction
POI density	number per area	Number of points of interest (POI; see above 15 rows) within a 5-kilometer buffer	24	Distance from closest point to each raster centroid calculated for prediction
MOHP dist. To stream divide	meters	Multi Order Hydrologic Position (MOHP), distance to stream divide for stream order 1, equal to the shortest distance to the stream plus the shortest distance to the Thiessen divide	25	
MOHP lateral position	meters	Multi Order Hydrologic Position (MOHP), lateral position for stream order 1, equal to the shortest distance to the stream divided by the distance to stream divide	25	
Road distance	meters	Distance to the nearest road	26	
Precipitation	Millimeters per year	Annual precipitation	27	
NLCD	unitless	One hot encoded National Land Cover Database (NLCD) categories for forest (mixed forest, evergreen forest, and deciduous forest); barren/grassland/herbaceous/shrub/scrub; wetlands	4	

		(herbaceous wetlands and woody wetlands); pasture/hay; developed, open space; developed, high intensity; developed, medium intensity; developed, low intensity	
Population density	Population per square kilometer	Population per square kilometer from 2010	[28]
Elevation	meters	Elevation at 1 Arc Second resolution	[29]
Lithology	unitless	One hot encoded lithology categories for felsic igneous; mafic igneous; undifferentiated pelitic; undifferentiated metamorphic; sulfidic schists; calcareous rocks; calcareous pelitic; calcareous metamorphic	[18]

Table S3. Wilcoxon one-sided signed rank test for differences between the 0-6 inch and 6-12 inch sampling intervals for compounds with detection frequencies exceeding 20% in both sampling intervals from the New Hampshire data. The *p*-values are calculated using both per- and polyfluoroalkyl substances (PFAS) concentrations (second column) and residual concentrations after regressing each PFAS compound against total organic carbon concentration (last column).

**Analyte abbreviation**	**Analyte name**	**Wilcoxon signed rank test *p*-value**	**Wilcoxon signed rank test *p*-value using PFAS residuals**
PFBA	Perfluorobutanoic acid	$p \leq 0.01$	$p \leq 0.05$
PFPeA	Perfluoropentanoic acid	$p \leq 0.01$	$p \geq 0.05$
PFHxA	Perfluorohexanoic acid	$p \leq 0.05$	$p \geq 0.05$
PFHpA	Perfluoroheptanoic acid	$p \leq 0.01$	$p \geq 0.05$
PFOA	Perfluorooctanoic acid	$p \leq 0.05$	$p \geq 0.05$
PFNA	Perfluorononanoic acid	$p \leq 0.001$	$p \leq 0.001$
PFDA	Perfluorodecanoic acid	$p \leq 0.001$	$p \leq 0.001$
PFUnA	Perfluoroundecanoic acid	$p \leq 0.001$	$p \leq 0.001$
PFDoA	Perfluorododecanoic acid	$p \leq 0.01$	$p \leq 0.05$
PFTrDA	Perfluorotridecanoic acid	$p \leq 0.001$	$p \leq 0.05$
PFBS	Perfluorobutane sulfonic acid	$p \leq 0.01$	$p \geq 0.05$
PFHxS	Perfluoropentane sulfonic acid	$p \leq 0.05$	$p \geq 0.05$
PFOS	Perfluorohexane sulfonic acid	$p \leq 0.001$	$p \leq 0.001$

References

1. Santangelo, L. M.; Tokranov, A. K.; Welch, S. M.; Schlosser, K. E. A.; Marts, J. M.; Drouin, A. F.; Ayotte, J. D.; Rousseau, A. E.; Harfmann, J. L. *Statewide survey of shallow soil concentrations of per- and polyfluoroalkyl substances (PFAS) and related chemical and physical data across New Hampshire, 2021*; U.S. Geological Survey data release; U.S. Geological Survey: Reston, VA, 2022; DOI: 10.5066/P9KG38B5.

2. Tokranov, A. K. *Data and model archive for shallow soil PFAS predictions in Maine, New Hampshire, and Vermont, 2026*; U.S. Geological Survey data release; U.S. Geological Survey: Reston, VA, 2026; DOI: 10.5066/P1K5IUJ6.

3. Scott, J. C. *Computerized stratified random site-selection approaches for design of a ground-water-quality sampling network*; U.S. Geological Survey Water-Resources Investigations Report 90-4101; U.S. Geological Survey: Reston, VA, 1990; DOI: 10.3133/wri904101.

4. Dewitz, J. *National Land Cover Database (NLCD) 2016 Products (ver. 2.0, July 2020)*; U.S. Geological Survey Data Release; U.S. Geological Survey: Reston, VA, 2019; DOI: 10.5066/P96HHBIE.

5. Soil Survey Staff *Field book for describing and sampling soils, Version 3.0*; USDA Natural Resources Conservation Service. U.S. Government Printing Office, 2012.

6. Kahn, L. *Determination of total organic carbon in sediment*; U.S. Environmental Protection Agency, Region II, Environmental Services Division: Edison, New Jersey, 1988.

7. *Methods for chemical analysis of water and wastes: Organic carbon, total, Method 415.1 (combustion or oxidation)*; U.S. Environmental Protection Agency, Office of Research and Development: Washington, DC, 1983.

8. Schindelbeck, R. R.; Moebius-Clune, B. N.; Moebius-Clune, D. J.; Kurtz, K. S.; van Es, H. M. *Cornell University Comprehensive Assessment of Soil Health Laboratory Standard Operating Procedures*; 2016.

9. Santangelo, L. M.; Welch, S. M.; Tokranov, A. K.; Schlosser, K. E. A.; Marts, J. M.; Lincoln, T. A.; Deyette, N. A. *Confirmatory sampling for per- and polyfluoroalkyl substances (PFAS) in shallow soils across New Hampshire, 2022*; U.S. Geological Survey data release; U.S. Geological Survey: Reston, VA, 2023; DOI: 10.5066/P9C0FAHD.

10. Sanborn, Head & Associates, Inc. *Background levels of PFAS and PAHs in Maine shallow soils*; 2022.

11. Zhu, W.; Roakes, H.; Zemba, S. G.; Badireddy, A. R. *PFAS background in Vermont shallow soils*; 2019.

12. Zhu, W.; Khan, K.; Roakes, H.; Maker, E.; Underwood, K. L.; Zemba, S.; Badireddy, A. R. Vermont-wide assessment of anthropogenic background concentrations of perfluoroalkyl substances in surface soils. *J. Hazard. Mater.* **2022,** *438*, 129479; DOI 10.1016/j.jhazmat.2022.129479.

13. Chaney, N. W.; Minasny, B.; Herman, J. D.; Nauman, T. W.; Brungard, C. W.; Morgan, C. L. S.; McBratney, A. B.; Wood, E. F.; Yimam, Y. POLARIS soil properties: 30-m probabilistic maps of soil properties over the contiguous United States. *Water Resour. Res.* **2019,** *55* (4), 2916-2938; DOI 10.1029/2018WR022797.

14. Flynn, R.; Tasker, G. *Generalized estimates from streamflow data of annual and seasonal ground-water-recharge rates for drainage basins in New Hampshire*; U.S. Geological Survey

Scientific Investigations Report 2004-5019; U.S. Geological Survey: Reston, VA, 2004; DOI: 10.3133/sir20045019.
15. Kim, J. J.; Ryan, P. C.; Schroeder, T.; Romanowicz, E.; Boutt, D.; Belaval, M.; Shanley, J. Four-dimensional characterization of a PFOA-contaminated fractured rock aquifer (FRA) in Bennington, Vermont, U.S.A. *Frontiers in Water* **2023,** *5*, DOI 10.3389/frwa.2023.1117780.
16. New Hampshire Department of Environmental Services Geodata Portal Saint-Gobain Consent Decree Outer Boundary. https://nh-department-of-environmental-services-open-data-nhdes.hub.arcgis.com/datasets/saint-gobain-consent-decree-outer-boundary/explore
17. New Hampshire Department of Environmental Services, Pages 59-71. https://www4.des.state.nh.us/OnestopPub/Air/330110016509262018TYPECT.pdf
18. Robinson Jr., G. R.; Kapo, K. E. *Generalized lithology and lithogeochemical character of near-surface bedrock in the New England region*; U.S. Geological Survey Open-File Report 03-225; U.S. Geological Survey: Reston, VA, 2003; DOI: 10.3133/ofr03225.
19. Ayotte, J. D.; Nielsen, M. G.; Robinson Jr, G.; Moore, R. B. *Relation of arsenic, iron, and manganese in ground water to aquifer type, bedrock lithogeochemistry, and land use in the New England coastal basins*; U.S. Geological Survey Water-Resources Investigations Report 99-4162; U.S. Geological Survey: Reston, VA, 1999; DOI: 10.3133/wri994162.
20. New Hampshire Department of Environmental Services NHDES PFAS Sampling Dashboard. https://nhdes.maps.arcgis.com/apps/dashboards/78fe1cb292af4cefbd49f281c43c658d
21. Miller, R. O.; Kissel, D. E. Comparison of soil pH methods on soils of North America. *Soil Sci. Soc. Am. J.* **2010,** *74* (1), 310-316; DOI 10.2136/sssaj2008.0047.
22. Pribyl, D. W. A critical review of the conventional SOC to SOM conversion factor. *Geoderma* **2010,** *156* (3), 75-83; DOI 10.1016/j.geoderma.2010.02.003.
23. National Atmospheric Deposition Program Annual NTN maps by year. https://nadp.slh.wisc.edu/maps-data/ntn-gradient-maps/
24. PFAS analytic tools. https://echo.epa.gov/trends/pfas-tools
25. Moore, R.; Belitz, K.; Arnold, T. L.; Sharpe, J. B.; Starn, J. J. *National Multi Order Hydrologic Position (MOHP) predictor data for groundwater and groundwater-quality modeling*; U.S. Geological Survey data release; U.S. Geological Survey: Reston, VA, 2019; DOI: 10.5066/P9HLU4YY.
26. U.S. Geological Survey Transportation. https://apps.nationalmap.gov/downloader/
27. PRISM Climate Group Parameter-elevation Regression on Independent Slopes (PRISM) Climate Group at Oregon State University, United States average annual precipitation, 1981-2010 (800m; ASCII GRID). http://www.prism.oregonstate.edu/normals/
28. Falcone, J. A. *U.S. block-level population density rasters for 1990, 2000, and 2010*; U.S. Geological Survey data release; U.S. Geological Survey: Reston, VA, 2022, 2016; DOI: 10.5066/F74J0C6M.
29. U.S. Geological Survey USGS 3D elevation program digital elevation model. https://apps.nationalmap.gov/downloader/
